# Extremophilic Bacteria Improve Germination, Early Vigor, and Oxidative-Stress Status in *Phaseolus vulgaris* Under Simulated Water Deficit

**DOI:** 10.3390/plants15193004

**Published:** 2026-09-30

**Authors:** Rodrigo Mora-Sanhueza, Cynthia Meza, Nicolas Flores-Castañón, Ricardo Tighe-Neira, Aparna Banerjee, Kattia Núñez-Montero, Valentina Vallejos, Patricio Arce-Johnson, Claudio Inostroza-Blancheteau

**Affiliations:** 1 Programa de Doctorado en Ciencias Agropecuarias, Facultad de Recursos Naturales, Universidad Católica de Temuco, Temuco 4781312, Chile; 2 Laboratorio de Fisiología y Biotecnología Vegetal, Departamento de Ciencias Agropecuarias y Acuícolas, Facultad de Recursos Naturales, Universidad Católica de Temuco, Temuco 4780000, Chile; 3 Functional Polysaccharides Research Group, Instituto de Ciencias Aplicadas, Facultad de Ingeniería, Universidad Autónoma de Chile, Talca 3460000, Chile; 4 Doctorado en Ciencias Biomédicas, Instituto de Ciencias Biomédicas, Facultad de las Ciencias de la Salud, Universidad Autónoma de Chile, Talca 3460000, Chile; 5 Departamento de Ciencias Agropecuarias y Acuícolas, Facultad de Recursos Naturales, Universidad Católica de Temuco, Rudecindo Ortega 02950, Temuco 4780000, Chile; 6 Núcleo de Investigación en Producción Alimentaria, Facultad de Recursos Naturales, Universidad Católica de Temuco, Temuco 4781312, Chile; 7 Instituto de Ciencias Aplicadas, Facultad de Ingeniería, Universidad Autónoma de Chile, Santiago 8580640, Chile; 8 ANID–Millennium Science Initiative Program–Millennium Nucleus in Data Science for Plant Resilience (Phytolearning), Santiago 8370186, Chile

**Keywords:** plant-growth-promoting bacteria (PGPB), bacterial consortium, extremophile, root architecture, bioinoculant, *Phaseolus vulgaris*, osmotic stress

## Abstract

Water deficit constrains common bean (*Phaseolus vulgaris* L.) production in the acidic Andisols of south-central Chile. We evaluated *Bacillus haynesii* CamB6 (thermotolerant, Chilean hydrothermal spring) and *Microbacterium* sp. Se63 (psychrotolerant, Antarctic soil), singly and as a two-strain consortium, for in vitro plant-growth-promoting traits and, via seed bacterization, on *P. vulgaris* cvs. Manteca and Señorita under simulated water deficit (PEG-8000, 0–15% *w*/*v*). The strains showed complementary in vitro profiles: Se63 solubilized more phosphate and produced siderophores and lipase, whereas CamB6 produced catalase, protease, HCN, and more indole-3-acetic acid. Responses in planta were stress- and cultivar-dependent. In the susceptible cultivar Manteca, Se63 alone increased shoot length and seedling dry weight under mild-to-moderate stress but did not rescue germination at 15% PEG, where it did not differ from the non-inoculated control; there, CamB6 was the most effective single strain (germination 31.0% to 52.0%). In the tolerant cultivar Señorita, Se63 alone produced the largest root-length gain under severe stress (100.4 to 208.6 cm) and the thickest roots, while the consortium rescued germination (65.0% to 93.0%), shoot length (+291%) and dry weight (+79%), attenuated lipid peroxidation and raised antioxidant capacity. Combining these extremophile strains therefore yields a stress-conditional, cultivar-dependent benefit in *P. vulgaris*.

## 1. Introduction

Water deficit is among the principal abiotic constraints limiting legume productivity worldwide, and its severity in south-central Chile, the country’s main common bean (*Phaseolus vulgaris* L.) production area, has intensified over the past decade. A persistent mega-drought has reduced regional rainfall by 20–40% and diminished Andean snowpack, and Chile is projected to rank among the thirty most water-scarce nations globally by 2040 under current climatic trends [1]. Because irrigation water is both scarcer and costlier under this scenario, agronomic strategies that increase stress resilience without increasing water input are of direct practical relevance to regional bean production.

Plant-growth-promoting bacteria (PGPB) are increasingly used as a low-input complement to conventional drought management. Documented mechanisms of PGPB-mediated drought alleviation include auxin (indole-3-acetic acid, IAA) biosynthesis that enhances root exploration, 1-aminocyclopropane-1-carboxylate (ACC) deaminase activity that limits stress-induced ethylene accumulation, solubilization of otherwise unavailable phosphate, siderophore-mediated iron acquisition, and modulation of osmolyte and antioxidant metabolism [2,3,4]. In *P. vulgaris* specifically, recent inoculation trials under deficit irrigation and simulated drought have reported improvements in relative water content, photosynthetic pigments, biomass, and oxidative-stress markers [5,6], and locally adapted rhizobial and PGPR strains have outperformed a reference commercial inoculant in sustaining bean growth under severe drought [7], supporting the premise that strain origin and local adaptation shape inoculation outcomes in this host.

Bacteria recovered from environmentally extreme habitats are an increasingly explored source of PGPB, on the premise that adaptation to physicochemical extremes co-selects for stress-relevant functional traits transferable to a plant host. A comparative genomic and functional screen of 29 *Microbacterium* isolates recovered ACC deaminase activity in 79% and combined IAA production with phosphate solubilization in 59% of strains tested, although annotated ACC deaminase genes were not recovered from the sequenced genomes, indicating that phenotypic potential in this genus is only partly resolved at the genome level [8]. *Microbacterium* strains have also been recovered, alongside other plant-beneficial isolates, from extreme Chilean environments including high-altitude Atacama Desert wetlands [9], and bacteria adapted to thermally extreme or arid sites, including geothermal isolates from the Atacama Desert and biofilm-forming rhizobacteria from semi-arid Iranian grasslands, have been shown to carry PGPB-relevant traits or to transfer stress tolerance to crop hosts under water deficit [10,11,12]. Building on this rationale, the present study used two previously characterized extremophile strains of contrasting thermal origin: *Bacillus haynesii* CamB6, isolated from the Campanario hydrothermal spring (Maule Region, Chile) and selected for its biofilm-, film-, and exopolysaccharide-forming capacity and stress-induced pigment production [13,14], and *Microbacterium* sp. Se63, isolated from Antarctic soil during a genomic and metabolomic bioprospecting survey [15] and selected as a cold-adapted representative of a genus with documented ACC deaminase activity, auxin and siderophore production, and phosphate-solubilizing potential [8].

Combining strains into a defined consortium can, in principle, extend single-strain benefit by pooling complementary functional traits, provided the combination does not simply reproduce the metabolic redundancy or interstrain competition that can offset this benefit under non-limiting conditions. Stress-conditional consortium benefit absent or negative under benign conditions but positive under water deficit has been documented directly in drought-stressed maize [16] and, with an explicit division of function between partner strains, in a two-strain alfalfa consortium under drought [17], and complementary rather than competitive agronomic performance has also been reported for a two-strain phosphate-solubilizing consortium in field-grown soybean [18]. Whether two given strains behave additively, competitively, or synergistically is therefore not a fixed property of consortium inoculation as such, but depends on the specific strain pair, the trait measured, and the stress level at which the comparison is made.

Despite this precedent, no previous study has, to our knowledge, tested a two-strain consortium built from phylogenetically and ecologically contrasting extremophiles, a thermophile and a psychrophile, on *P. vulgaris* under simulated water deficit. The objectives of this study were, first, to characterize the in vitro plant-growth-promoting and stress-tolerance traits of *B. haynesii* CamB6, *Microbacterium* sp. Se63, and their consortium’s phosphate solubilization, IAA production, and tolerance to PEG-8000-induced osmotic stress and, second, to evaluate seed bacterization with each strain alone and as a consortium on germination, early vigor, root architecture, and oxidative-stress-related biochemistry in two *P. vulgaris* cultivars of contrasting intrinsic tolerance (Manteca and Señorita) under simulated water deficit. Because CamB6 and Se63 display largely non-overlapping trait profiles, we hypothesized that the consortium would outperform the mean of its single-strain components specifically under moderate-to-severe osmotic stress, when complementary trait contribution outweighs the cost of functional redundancy, conferring no added or even negative benefit under non-stressed conditions.

## 2. Results

The plant-growth-promoting and stress-tolerance potential of *Bacillus haynesii* CamB6 and *Microbacterium* sp. Se63 was characterized in vitro (Section 2.1, Section 2.2, Section 2.3 and Section 2.4) before evaluating their effect, singly and as a two-strain consortium, on *Phaseolus vulgaris* cv. Manteca and cv. Señorita under combined seed bacterization and simulated water deficit (Section 2.5.1, Section 2.5.2, Section 2.5.3, Section 2.5.4, Section 2.5.5 and Section 2.5.6).

### 2.1. Screening of Plant-Growth-Promoting Traits

A qualitative screen of 10 in vitro plant-growth-promoting and enzymatic traits (Table 1) showed a complementary, largely non-overlapping profile between the two strains. *Microbacterium* sp. Se63 was positive for phosphate solubilization, siderophore production, and lipase activity, all absent in *Bacillus haynesii* CamB6, whereas CamB6 was positive for catalase, protease, and HCN production, all absent in Se63; both strains showed comparable ammonia production, mucoid phenotype, and N_2_ fixation, and amylase activity was scored equally for both (Table 1). Representative plate assays for phosphate solubilization, N_2_ fixation, HCN production, siderophore production, and ammonia production are shown in Figure S1.

### 2.2. Phosphate Solubilization

**Figure 1:**
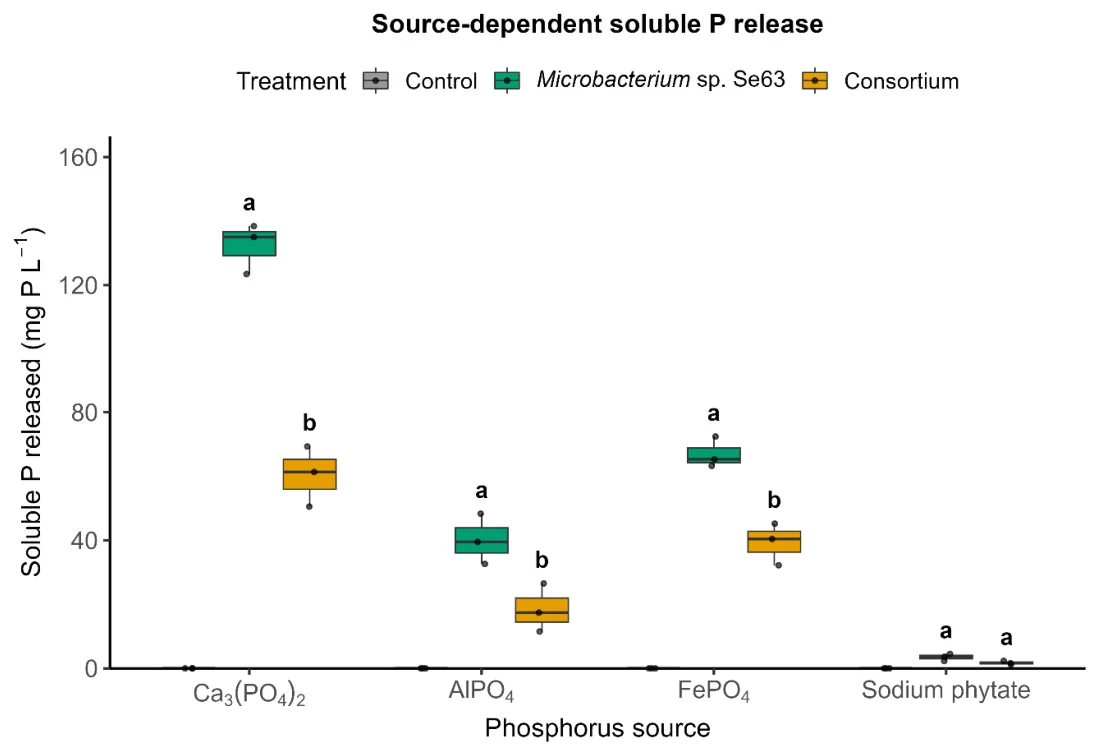
In vitro phosphate mobilization by *Microbacterium* sp. Se63 and the CamB6 + Se63 consortium from four phosphorus sources. Distribution of soluble P concentrations (mg P L^−1^) in the non-inoculated control, *Microbacterium* sp. Se63, and the CamB6 + Se63 consortium. Boxplots show the median, interquartile range, and individual observations (*n* = 3). Box colour identifies the bacterial treatment as given in the legend; the grey boxes correspond to the non-inoculated control, in which soluble P release remained at or near the detection limit, so they lie on the *x*-axis. Different lowercase letters within each P source indicate significant differences between bacterial treatments according to Tukey’s HSD test (α = 0.05).

The ability of *Microbacterium* sp. Se63 and the CamB6 + Se63 consortium to mobilize P from three sparingly soluble mineral phosphates, tricalcium phosphate, aluminum phosphate, and iron phosphate, and from sodium phytate as an organic P source was assessed in liquid NBRIP medium (Figure 1 and Figure S2); CamB6 alone was not included in this quantitative assay because the qualitative screen (Section 4.2; Table 1) had already shown it lacks detectable phosphate-solubilizing activity. Soluble P release was significantly affected by P source, bacterial treatment, and their interaction (two-way ANOVA, *p* < 0.001 in all cases; ηp^2^ ≥ 0.90). Mean soluble P release varied approximately 37-fold among sources, from 96.34 mg P L^−1^ for tricalcium phosphate to 2.62 mg P L^−1^ for sodium phytate. *Microbacterium* sp. Se63 released significantly more soluble P than the consortium from tricalcium phosphate, aluminum phosphate, and iron phosphate, with the largest difference observed for tricalcium phosphate (132.29 ± 7.84 vs. 60.40 ± 9.43 mg P L^−1^). No significant difference between bacterial treatments was detected for sodium phytate.

### 2.3. Indole-3-Acetic Acid Production

**Figure 2:**
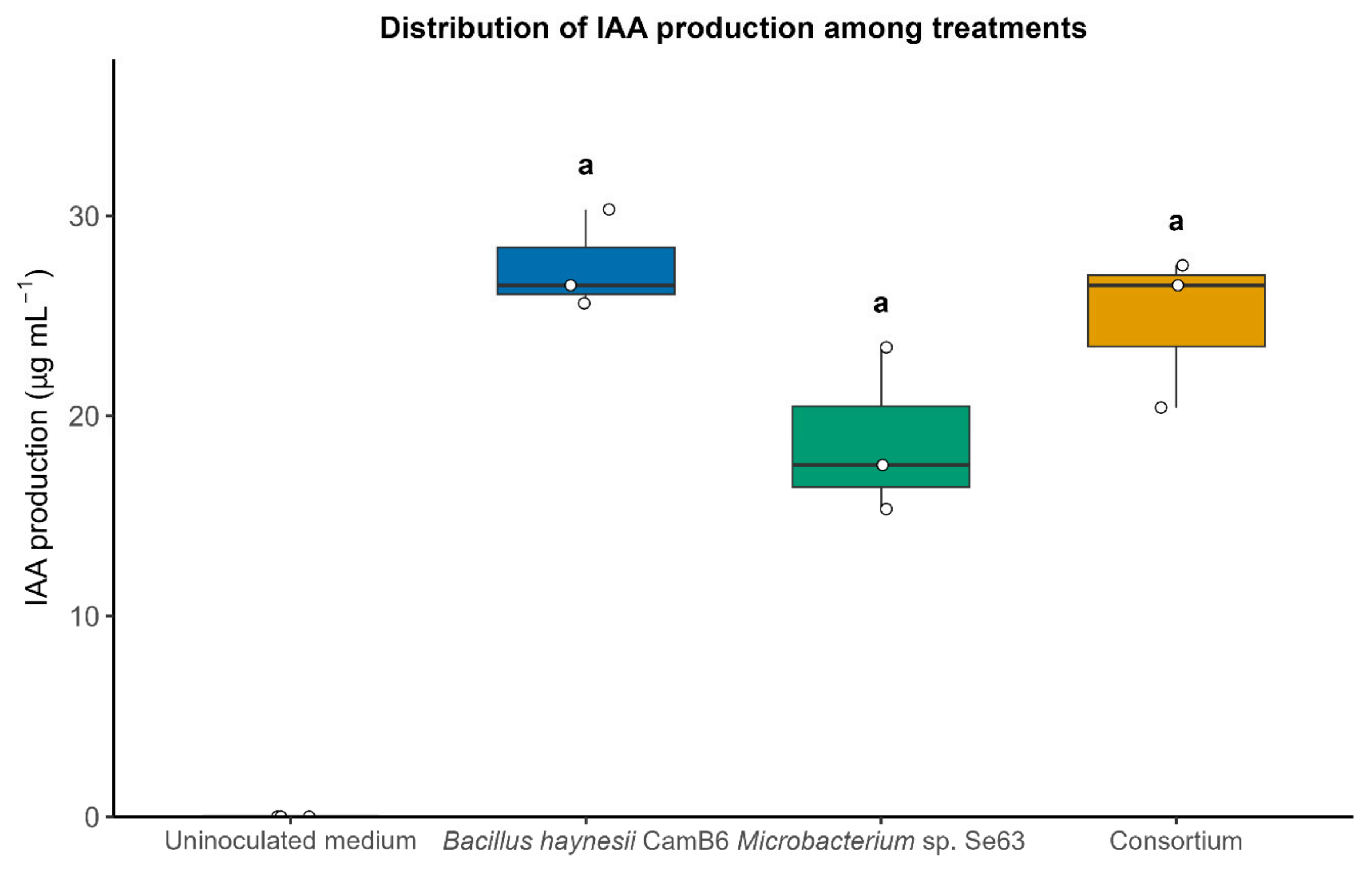
In vitro indole-3-acetic acid (IAA) production by *Bacillus haynesii* CamB6, *Microbacterium* sp. Se63 and their consortium. Salkowski colorimetric assay, semi-synthetic medium with 5 mM L-tryptophan, 7 days, 27 °C. Boxplots show the median, interquartile range and individual observations, with the white point indicating the mean (*n* = 3); box colour identifies the bacterial treatment as given on the *x*-axis. Different lowercase letters above the boxes indicate significant differences among treatments (one-way ANOVA followed by Tukey’s HSD, α = 0.05); the three inoculated treatments share the same letter and therefore did not differ significantly from one another. No IAA was detected in the uninoculated medium, whose observations lie on the *x*-axis.

Indole-3-acetic acid production was compared among *Bacillus haynesii* CamB6, *Microbacterium* sp. Se63, and their consortium. Additionally, the medium was used as a negative control (Figure 2). Production was numerically highest for CamB6 (27.49 ± 2.49 µg mL^−1^), intermediate for the consortium (24.83 ± 3.85 µg mL^−1^) and lowest for Se63 (18.76 ± 4.19 µg mL^−1^), but the omnibus effect fell short of significance at this sample size (one-way ANOVA, *p* = 0.060, η^2^ = 0.609); the CamB6-vs.-Se63 contrast showed a large effect size (Hedges’ g = 2.03) that did not survive Tukey correction (*p* = 0.056).

### 2.4. Bacterial Tolerance to PEG-8000-Induced Osmotic Stress

**Figure 3:**
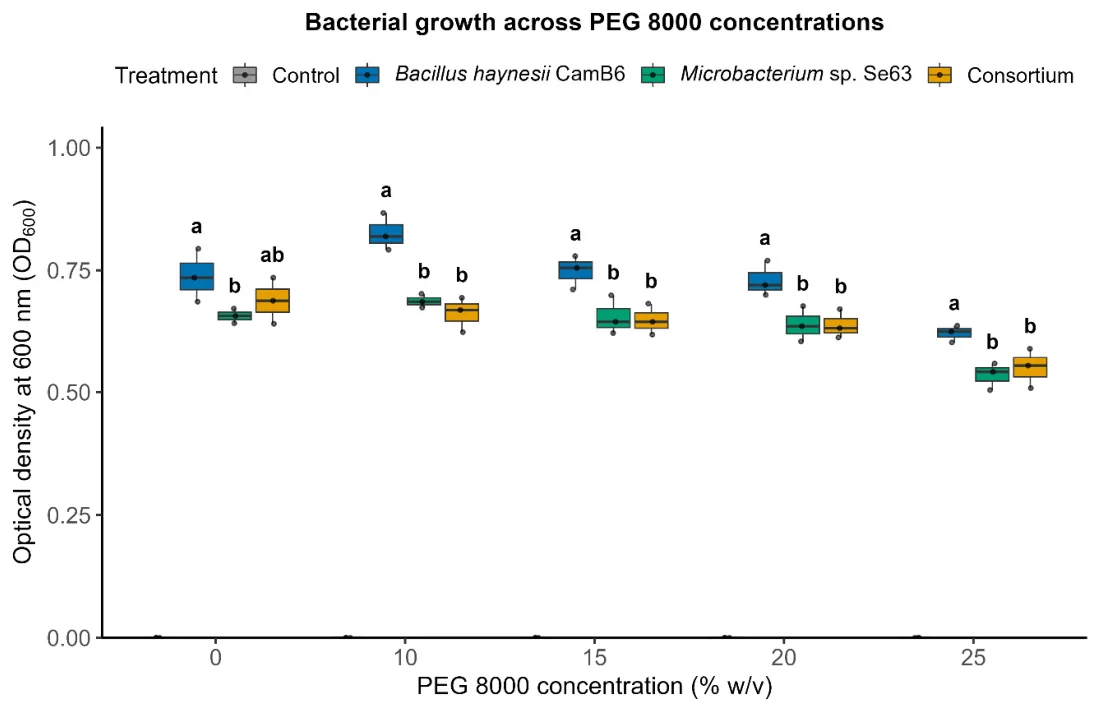
In vitro bacterial tolerance to PEG-8000-induced osmotic stress. Culture turbidity (OD_600_) across five PEG-8000 concentrations, 0–25% *w*/*v*, 24 h, 27 °C. Boxplots show the median, interquartile range and individual observations (*n* = 3); box colour identifies the bacterial treatment as given in the legend, the grey boxes corresponding to the non-inoculated control, whose OD_600_ values lie on the *x*-axis. Different lowercase letters within each PEG-8000 concentration indicate significant differences among bacterial treatments (two-way factorial ANOVA followed by Tukey’s HSD, α = 0.05).

Tolerance of the three bacterial treatments to PEG-8000-induced osmotic stress was assessed as culture turbidity across five concentrations, 0–25% *w*/*v* (Figure 3). PEG level and treatment identity both significantly affected turbidity (two-way ANOVA, *p* < 0.001 in both cases; η^2^p = 0.72–0.78), with no significant interaction (*p* = 0.354). *Bacillus haynesii* CamB6 maintained higher turbidity than Se63 and the consortium across the entire gradient, and growth declined only from 20% PEG onward, reaching its lowest value at 25% (−18.0% relative to the unstressed control).

### 2.5. Seed Bacterization and Germination Under PEG-8000-Induced Osmotic Stress

Bacterial effects on seed germination, early seedling vigor, root architecture, and seedling biochemistry were evaluated in both cultivars under a complete cultivar × PEG-8000 (0–15%) × inoculation factorial design (six biological replicates per cell, *N* = 192). Free proline content was analyzed by conventional three-way ANOVA after log-transformation; the remaining variables were evaluated by the Aligned Rank Transform. Post hoc comparisons used Tukey’s HSD; effect sizes and Bayes factors are reported for headline contrasts. All two- and three-way interactions were tested; interactions that did not reach significance are not reported individually, and the corresponding main-effect or lower-order comparisons are presented instead.

#### 2.5.1. Biochemical Determinations in Germinating Seeds

Total phenolic content (TPC) and DPPH radical-scavenging activity were measured in germinating seeds at 24, 48, and 72 h post-inoculation across the same cultivar × PEG × inoculation × sampling-time structure described above (Table S1), with cultivar included as a crossed factor. TPC varied strongly with germination time (*p* < 0.001, η^2^p = 0.978), with a significant Cultivar × Time interaction underlying this pattern: this time course differed between cultivars; non-inoculated ’Manteca’ peaked at 48 h (27.4 mg GAE g^−1^ FW) before declining by 72 h, while ’Señorita’ followed a flatter time course over the same window (Table S1). PEG stress significantly increased TPC at a given time point in both cultivars (η^2^p = 0.726). Inoculation raised TPC at 24 h only (Table S1), most strongly with the bacterial consortium, whose advantage scaled with PEG severity; from 48 h onward (Table S1), the non-inoculated control became the highest-TPC treatment in both cultivars, and the consortium’s advantage over the control reversed from +13.9% at 24 h to roughly −20% from 48 h onward.

DPPH activity followed the same temporal and PEG pattern as TPC (Table S1), with an even stronger, time-shifted crossover with inoculation (Time × Inoculation interaction, *p* < 0.001, η^2^p = 0.805): the consortium produced the largest radical-scavenging activity in both cultivars at 24 h (Table S1; e.g., +80.0% over the control in ’Señorita’ at PEG 15%), an advantage that reversed by 72 h, when the non-inoculated control showed the highest activity in most cells (Table S1). TPC and DPPH were positively correlated overall (r = 0.425, *p* < 0.001), more strongly within each cultivar (r = 0.618–0.719).

Total phenolic content and DPPH radical-scavenging activity data for germinating seeds are presented in Table S1.

#### 2.5.2. Confocal Laser Scanning Microscopy of Root–Bacteria Association

**Figure 4:**
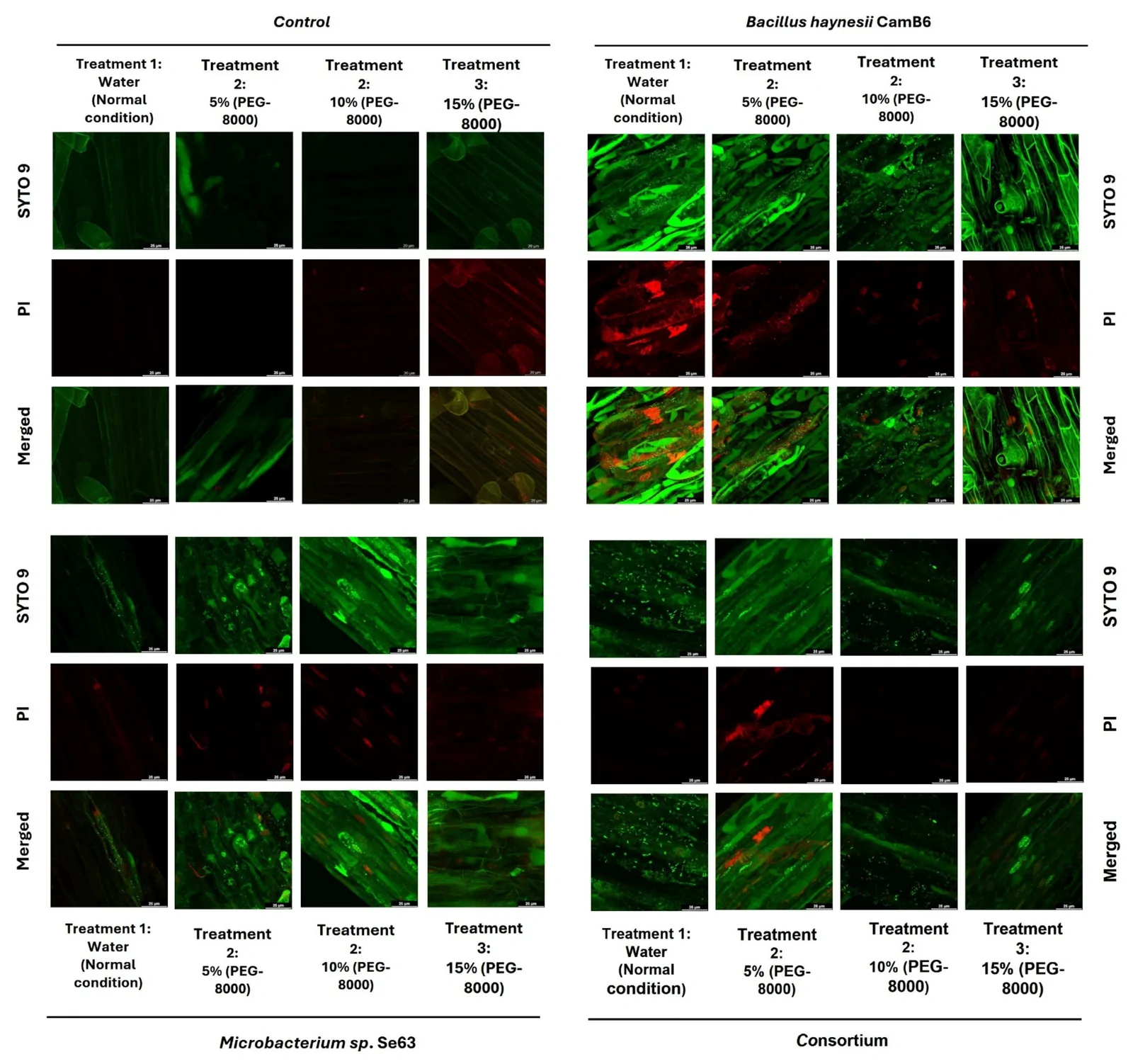
Confocal laser-scanning microscopy of root-associated bacterial viability (cv. Manteca). SYTO9 (green, viable cells)/propidium iodide (PI; red, membrane-compromised cells) LIVE/DEAD staining, 60× magnification, 7 days post-inoculation, across four inoculation treatments and four PEG-8000 levels (0–15%). Representative images from independent fields. All three inoculated treatments show markedly more extensive SYTO9 signal than the control, confirming root colonization. CamB6 shows the most extensive PI signal, while the consortium combines the densest SYTO9 signal with the most limited PI signal.

**Figure 5:**
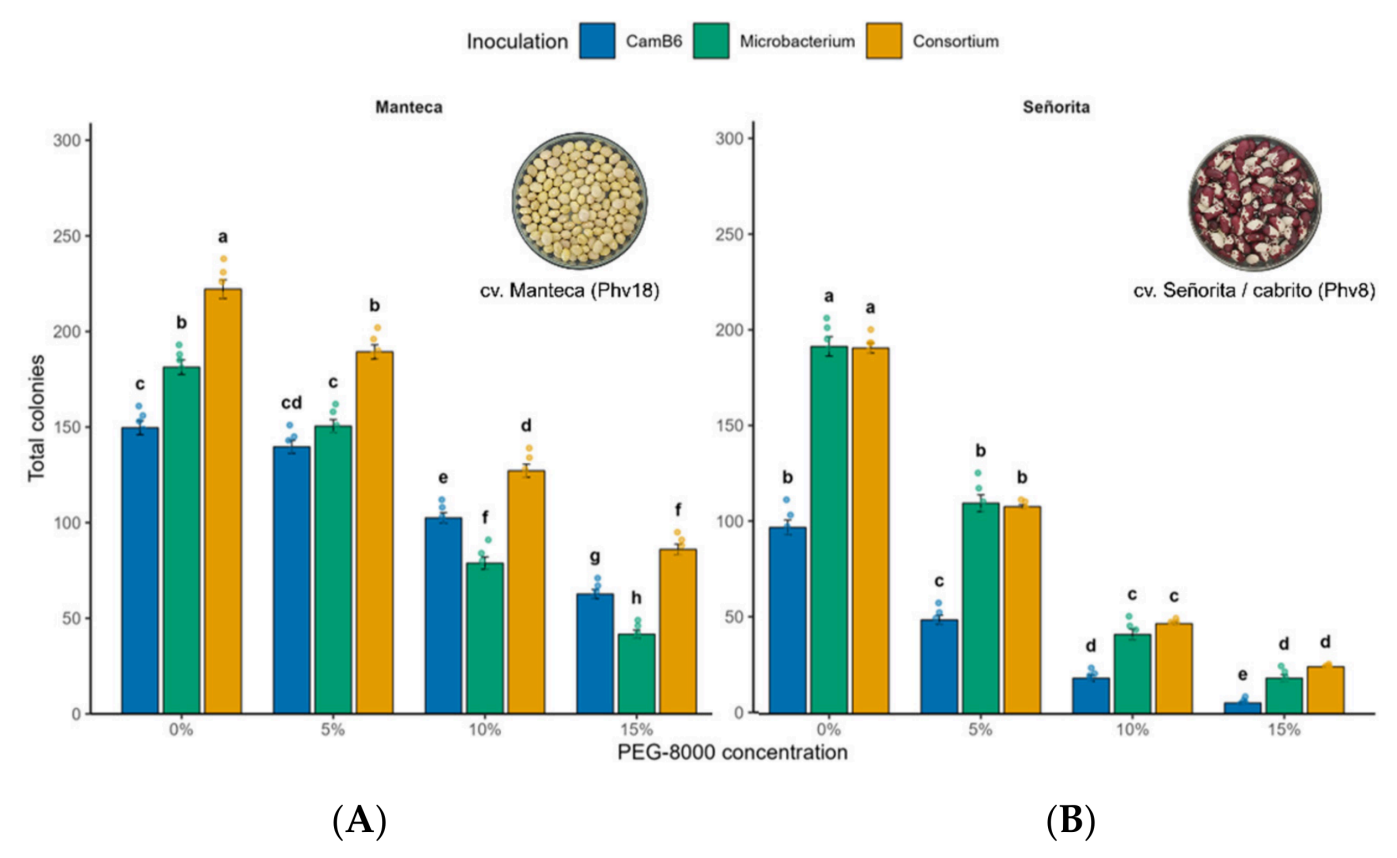
Root colonization: strain-identifying reference plates and culturable bacterial recovery from root washes. (**A**) Pure-culture reference plates and root-wash dilution plates for cv. Manteca and cv. Señorita, by treatment and PEG-8000 level. (**B**) Total colony counts (CFU) from root washes 7 days post-inoculation, by treatment and PEG-8000 level. Data are mean ± SD. In (**B**), bar colour identifies the inoculation treatment as given in the legend, and different lowercase letters indicate significant differences among inoculation × PEG-8000 combinations within each cultivar (Tukey’s HSD, α = 0.05); letters are assigned independently in each cultivar panel.

Root-associated bacterial colonization was assessed 7 days post-inoculation by confocal microscopy in cv. Manteca (SYTO9/propidium iodide viability staining; Figure 4) and by culturable recovery from root washes in both cultivars (Figure 5). All three inoculated treatments showed markedly more extensive SYTO9 (viable-cell) signal than the non-inoculated control across the PEG-8000 gradient, confirming root colonization by all three strains. *Bacillus haynesii* CamB6 showed the most extensive propidium iodide signal, indicating a larger membrane-compromised fraction within its root-associated population, whereas the consortium combined the densest SYTO9 signal with the most limited propidium iodide signal of the three treatments (Figure 4).

Pure-culture reference plates showed the distinct colony morphology of pigmented CamB6 versus cream/white Se63 used to identify each strain on root-wash plates (Figure 5A); the full photographic record is provided in Figure S3. Culturable bacterial recovery from root washes declined with increasing PEG-8000 concentration in both cultivars and all three treatments (Figure 5B), paralleling the osmotic-stress effects on in vitro bacterial growth (Figure 3 and Table S1) and on whole-plant growth (Section 2.5.3, Section 2.5.4 and Section 2.5.5). The consortium produced the highest, or joint-highest, colony count at every PEG level in both cultivars. The two single strains reversed rank between cultivars: CamB6 recovered better than Se63 in cv. Manteca at 10–15% PEG, but consistently worse in cv. Señorita, with near-complete loss of recoverable CamB6 at 15% PEG in that cultivar, mirroring the cultivar-specific inoculation responses already observed for phenolic content and DPPH radical-scavenging activity during germination (Table S1).

#### 2.5.3. Germination and Early-Vigor Indices

**Figure 6:**
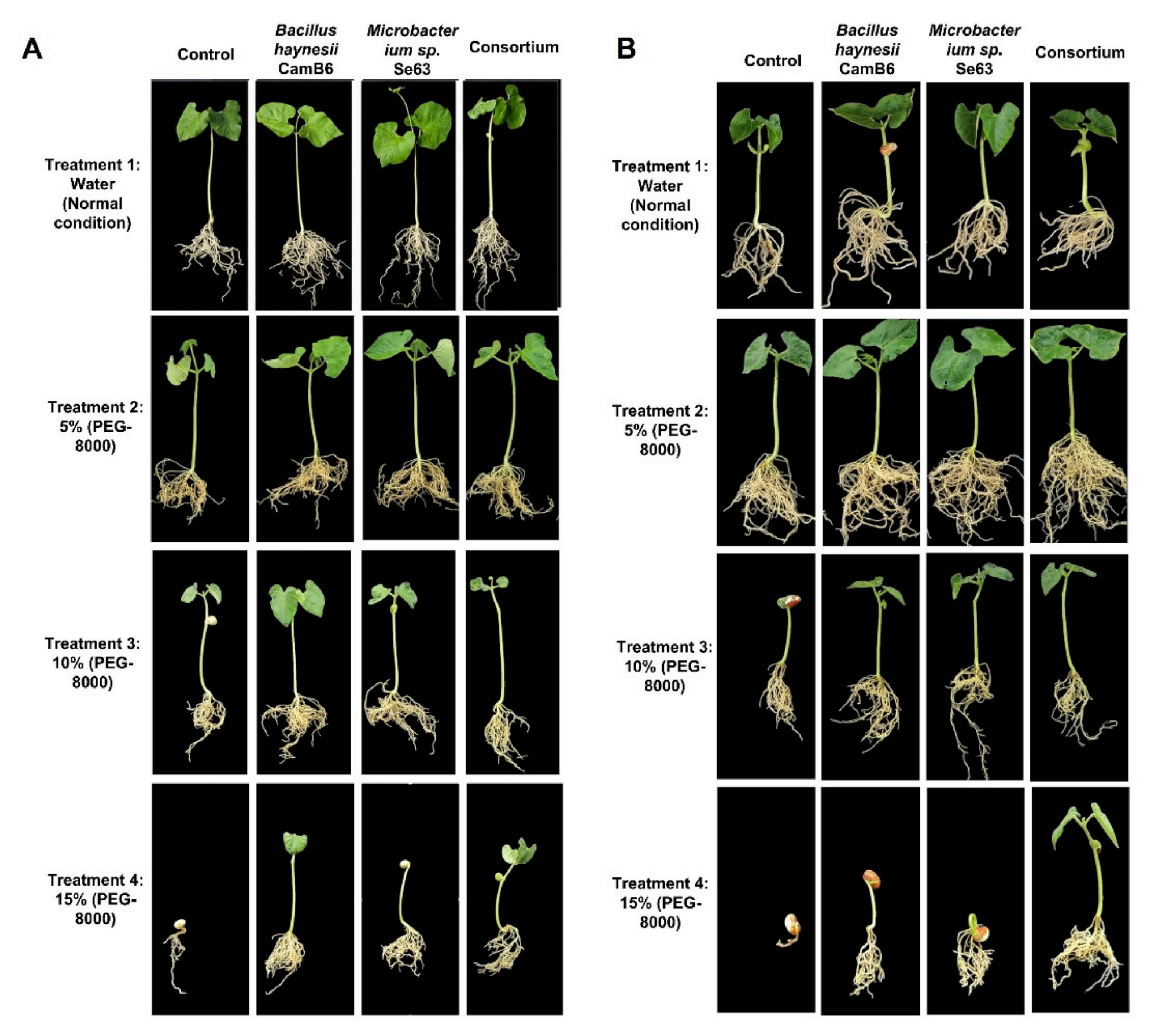
Representative whole-seedling and root phenotypes 12 days after inoculation (BBCH stage 12). Cv. Manteca (**A**) and cv. Señorita (**B**), by inoculation treatment and PEG-8000 level (0–15%). Non-inoculated seedlings show reduced shoot and root development with increasing PEG-8000 concentration, most severe at 15%, while inoculated seedlings retain more extensive growth at the same stress levels.

Representative whole-seedling photographs 12 days after inoculation illustrate this pattern: non-inoculated seedlings showed reduced shoot and root development with increasing PEG-8000 concentration in both cultivars, while inoculated seedlings, particularly with the consortium, retained more extensive root systems and shoot growth under the same stress (Figure 6).

**Figure 7:**
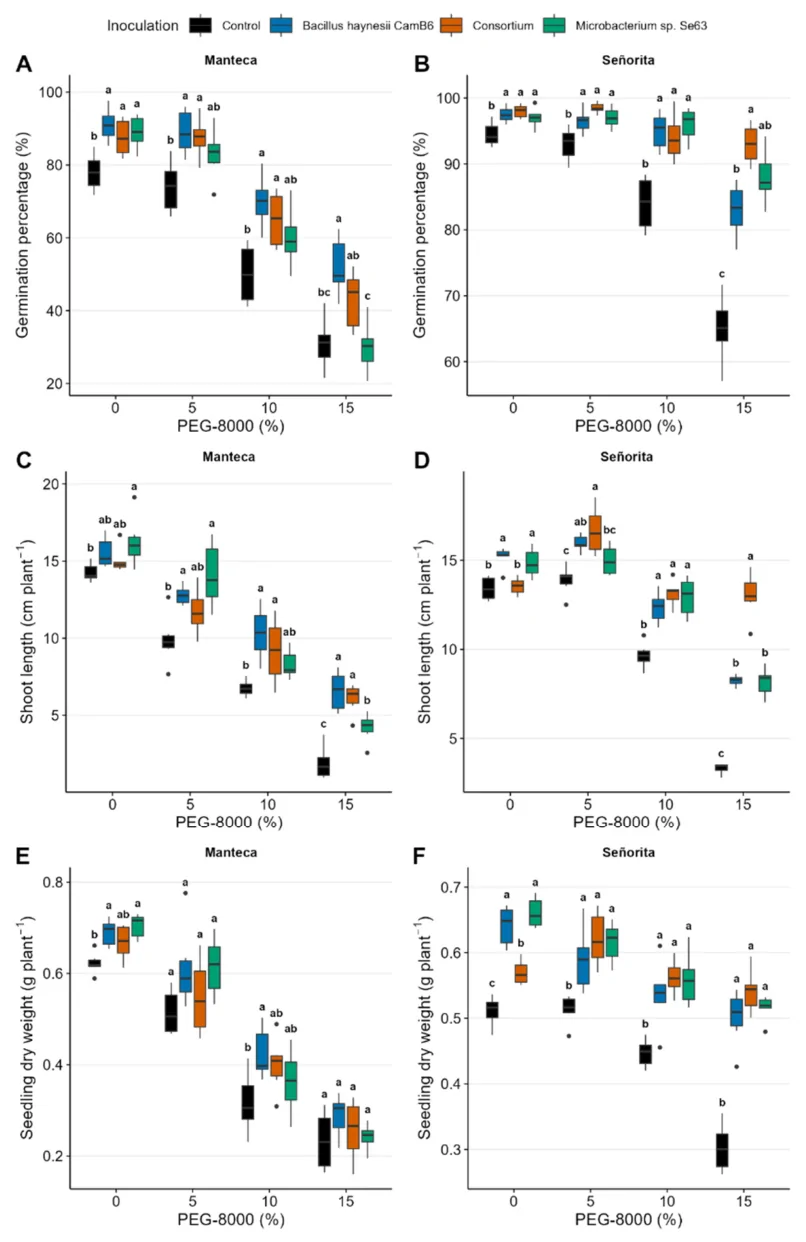
Germination and early seedling growth under combined bacterization and PEG-8000-induced osmotic stress. Germination percentage ((**A**) Manteca; (**B**) Señorita), shoot length ((**C**) Manteca; (**D**) Señorita), and seedling dry weight ((**E**) Manteca; (**F**) Señorita), 12 days after inoculation. Data are mean ± SD (*n* = 6). Tukey’s HSD, compact-letter display, α = 0.05; different lowercase letters indicate significant differences among inoculation treatments within each PEG level. All three variables declined with PEG-8000 concentration in non-inoculated controls and were significantly rescued by inoculation, most consistently in cv. Señorita.

Germination percentage declined with PEG-8000 concentration in both cultivars, but more steeply in ’Manteca’ than in ’Señorita’ (Cultivar × PEG interaction, *p* < 0.001, η^2^ = 0.690; Figure 7A,B). In non-inoculated controls, germination fell from 78.0% to 31.0% in ’Manteca’ and from 94.5% to 65.0% in ’Señorita’ across the same PEG gradient, indicating that ’Señorita’ is intrinsically more tolerant at germination. Inoculation significantly improved germination under stress in both cultivars (*p* < 0.001, η^2^ = 0.532), most notably at PEG 15%, where CamB6 raised germination to 52.0% in ’Manteca,’ and the consortium raised it to 93.0% in ’Señorita’.

Shoot length (Figure 7C,D) and seedling dry weight (Figure 7E,F) showed the same stress-driven decline and inoculation rescue, with a cultivar asymmetry in its robustness: in ’Señorita’, the consortium significantly improved dry weight (+79.0%) and shoot length (+291.4%) even at the most severe stress level, whereas in ’Manteca’ CamB6’s rescue was significant at moderate stress (+34.0% dry weight at PEG 10%) but did not reach significance at PEG 15% (+23.8%, *p* = 0.116).

**Figure 8:**
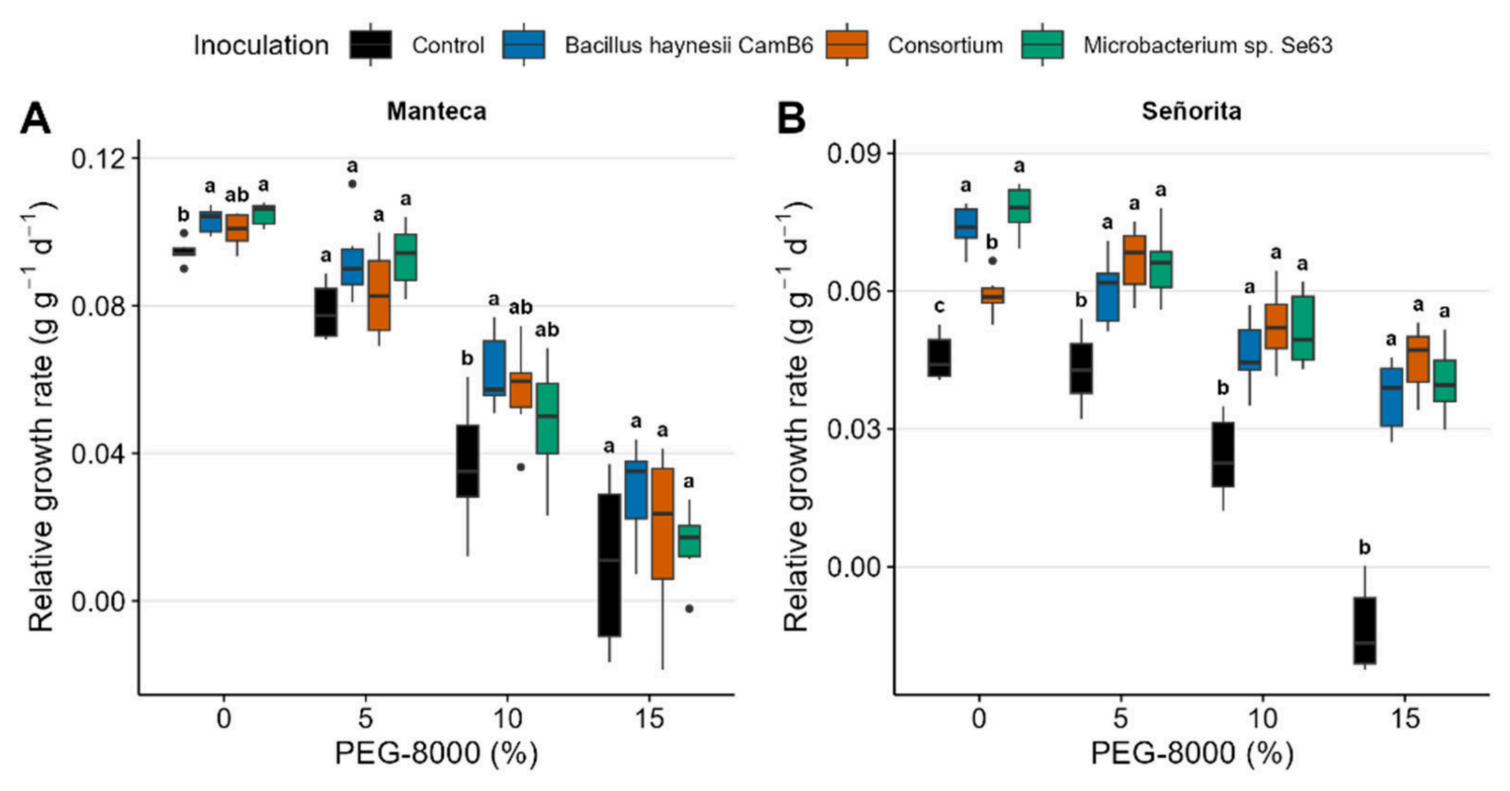
Relative growth rate under combined bacterization and PEG-8000-induced osmotic stress. Cv. Manteca (**A**) and cv. Señorita (**B**), 12 days after inoculation. Data are mean ± SD (*n* = 6). Tukey’s HSD, compact-letter display, α = 0.05; different lowercase letters indicate significant differences among inoculation treatments within each PEG level. Growth rate became negative in stressed, non-inoculated ‘Señorita’ seedlings and was restored to a positive value by consortium inoculation.

Relative growth rate mirrored this pattern (Figure 8A,B): it became negative in stressed, non-inoculated ’Señorita’ seedlings (−0.014 g g^−1^ d^−1^ at PEG 15%), indicating net biomass loss, and was restored to a positive value by consortium inoculation (+0.045 g g^−1^ d^−1^), while ’Manteca’ controls retained a low positive rate under the same stress.

#### 2.5.4. Image-Based Analysis of Root System Architecture

**Figure 9:**
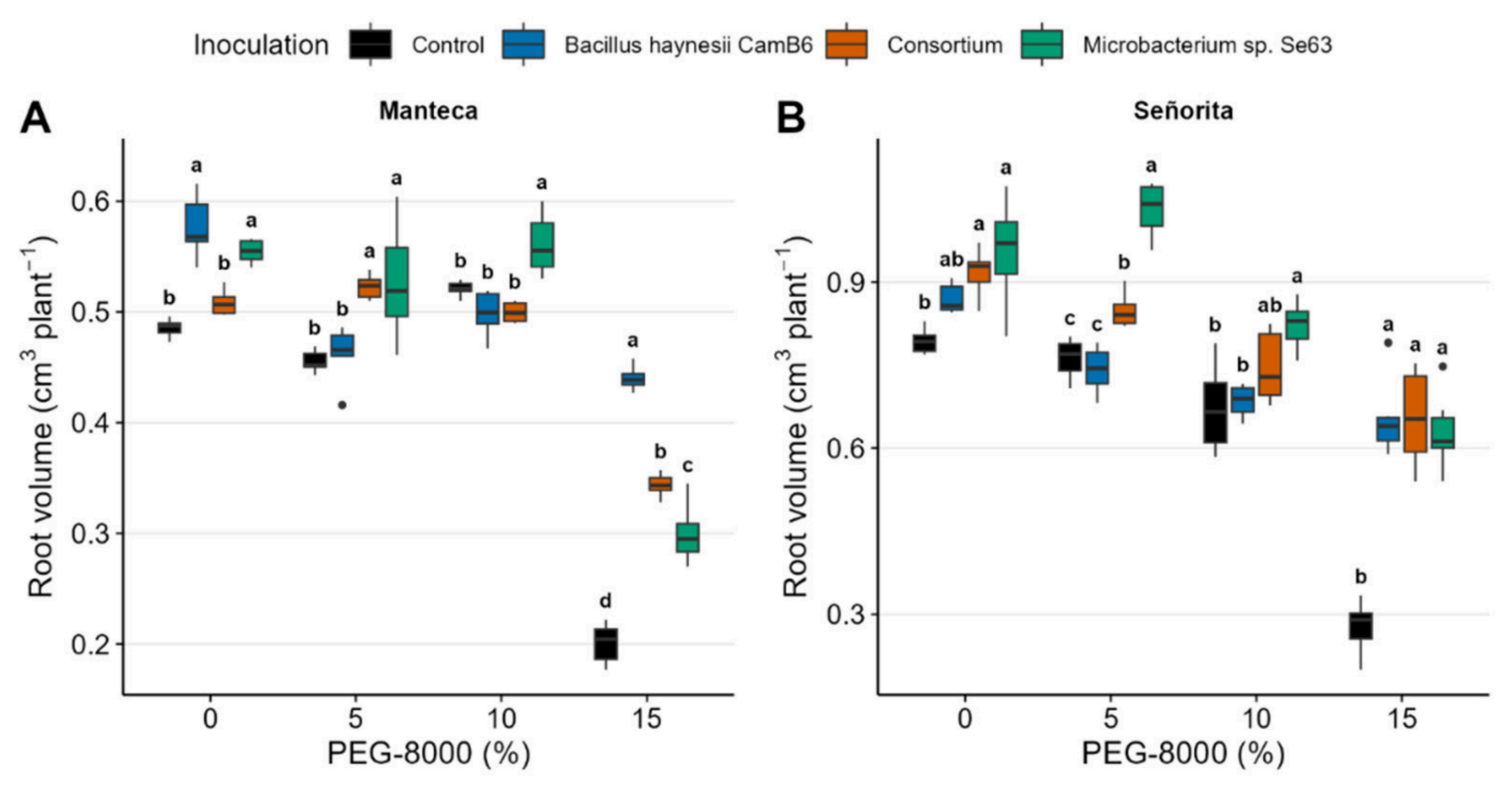
Root volume under combined bacterization and PEG-8000-induced osmotic stress. Cv. Manteca (**A**) and cv. Señorita (**B**), WinRHIZO image analysis, 12 days after inoculation. Data are mean ± SD (*n* = 6). Tukey’s HSD, compact-letter display, α = 0.05; different lowercase letters indicate significant differences among inoculation treatments within each PEG level.

Root length, surface area, and volume all declined with PEG concentration in non-inoculated controls of both cultivars, while average root diameter followed a distinct, more inoculation-dependent trajectory (Cultivar × PEG interaction, *p* < 0.001, η^2^ = 0.451). Root volume (Figure 9A,B) declined by 58.6% (’Manteca’) and 65.0% (’Señorita’) from PEG 0 to 15% in non-inoculated controls and was substantially rescued by inoculation, with CamB6 approximately doubling root volume in stressed ’Manteca’ (Figure 9A) and the consortium doing so in ’Señorita’ (Figure 9B). Root length and surface area showed an analogous decline and rescue (root length, consortium: 61.6 to 156.3 cm in stressed ’Manteca’; 100.4 to 208.6 cm with Se63 in ’Señorita’). Average root diameter was essentially uncorrelated with the other architectural traits (|*r*| ≤ 0.16) and responded to inoculation in a strain-specific manner, with CamB6 associated with thicker roots in ’Manteca’ and Se63 in ’Señorita’ under severe stress.

#### 2.5.5. Biochemical Determinations in Seedling Tissues

**Figure 10:**
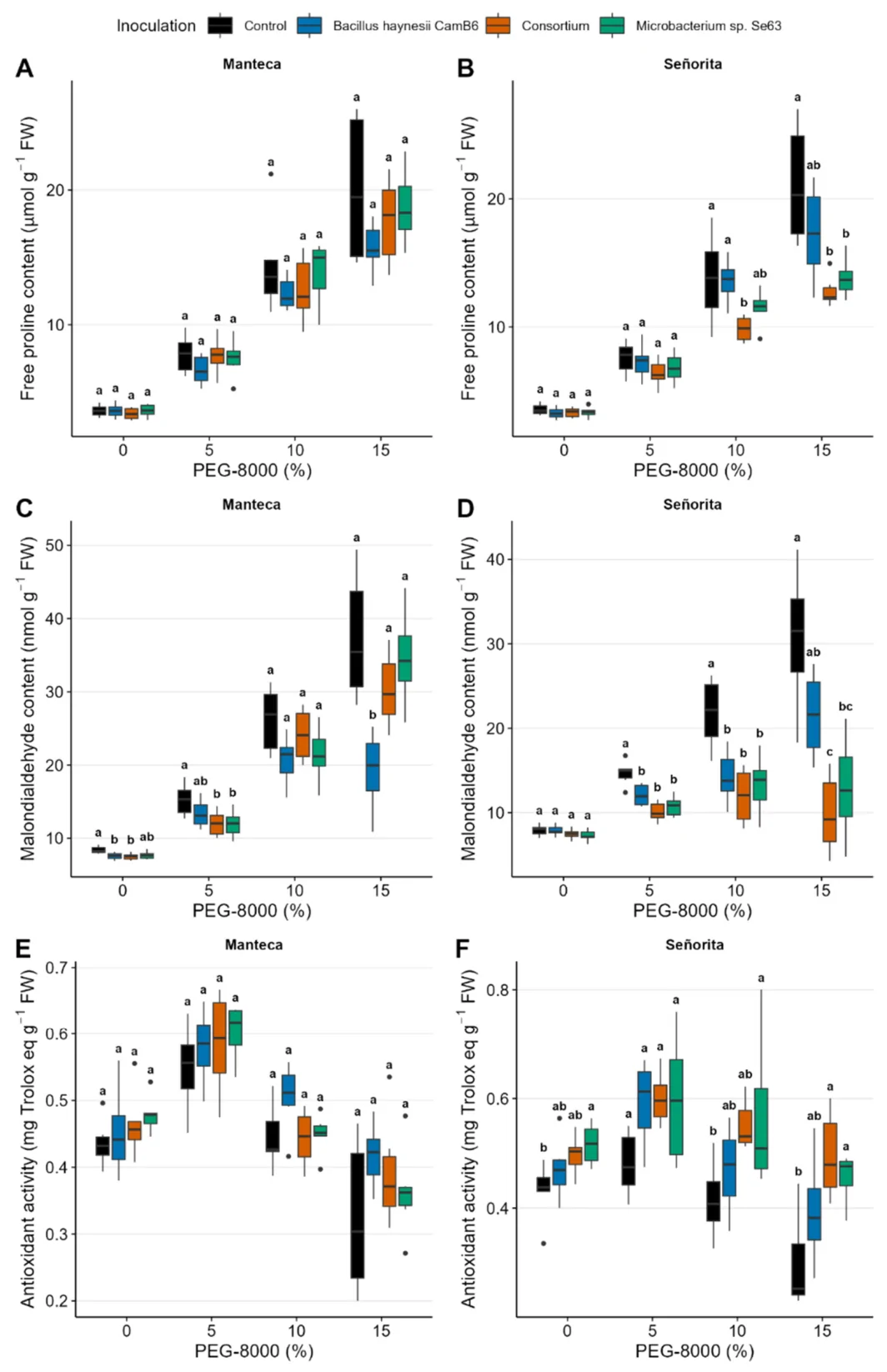
Osmotic adjustment, lipid peroxidation, and antioxidant activity in seedling tissue. Free proline ((**A**) Manteca; (**B**) Señorita), malondialdehyde/MDA content ((**C**) Manteca; (**D**) Señorita), and Trolox-equivalent antioxidant activity ((**E**) Manteca; (**F**) Señorita), 12 days after inoculation, on a dry-weight basis. Data are mean ± SD (*n* = 4). Tukey’s HSD, compact-letter display, α = 0.05; different lowercase letters indicate significant differences among inoculation treatments within each PEG level.

**Figure 11:**
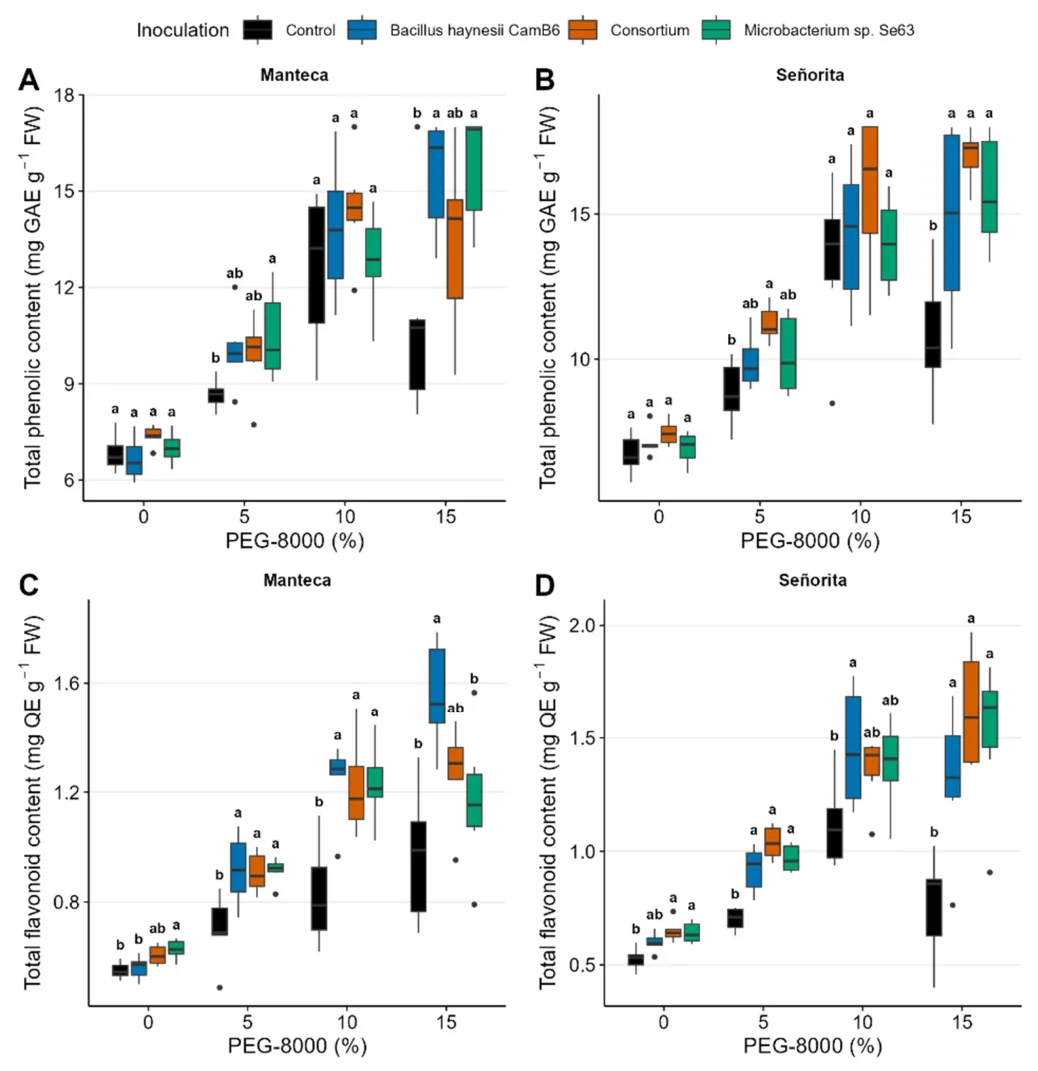
Total phenolic and flavonoid content in seedling tissue. Total phenolic content ((**A**) Manteca; (**B**) Señorita) and total flavonoid content ((**C**) Manteca; (**D**) Señorita), 12 days after inoculation, on a dry-weight basis. Data are mean ± SD (*n* = 4). Tukey’s HSD, compact-letter display, α = 0.05; different lowercase letters indicate significant differences among inoculation treatments within each PEG level.

Free proline content (Figure 10A,B) increased steeply with PEG concentration in both cultivars (three-way ANOVA, main effect of PEG, *p* < 0.001), rising more than four-fold from PEG 0 to 15% in non-inoculated controls. A significant Cultivar × Inoculation interaction underlay a modest, cultivar-dependent inoculation effect: in ’Señorita’ at PEG 10%, the consortium significantly lowered proline relative to the stressed control, while in ’Manteca’ inoculation did not alter proline at any PEG level. Malondialdehyde (MDA; Figure 10C,D), a marker of lipid peroxidation, also rose sharply with stress severity in non-inoculated controls of both cultivars. Inoculation significantly attenuated MDA accumulation in ’Señorita’ CamB6, lowering MDA by 34.7% at PEG 10% and 29.7% at PEG 15%, but not in ’Manteca’ at any PEG level, mirroring the cultivar asymmetry already noted for biomass and growth rate. Trolox-equivalent antioxidant activity (Figure 10E,F) increased with inoculation only in ’Señorita’ (consortium at PEG 15%, +66.9% over control, *p* = 0.002); no inoculation effect on this variable was detected in ’Manteca’ at any PEG level. Total phenolic (Figure 11A,B) and flavonoid content (Figure 11C,D) in seedling tissue increased with PEG severity in non-inoculated controls of both cultivars and were further increased by inoculation under stress.

#### 2.5.6. Multivariate Structure of the Phenotypic Response

**Figure 12:**
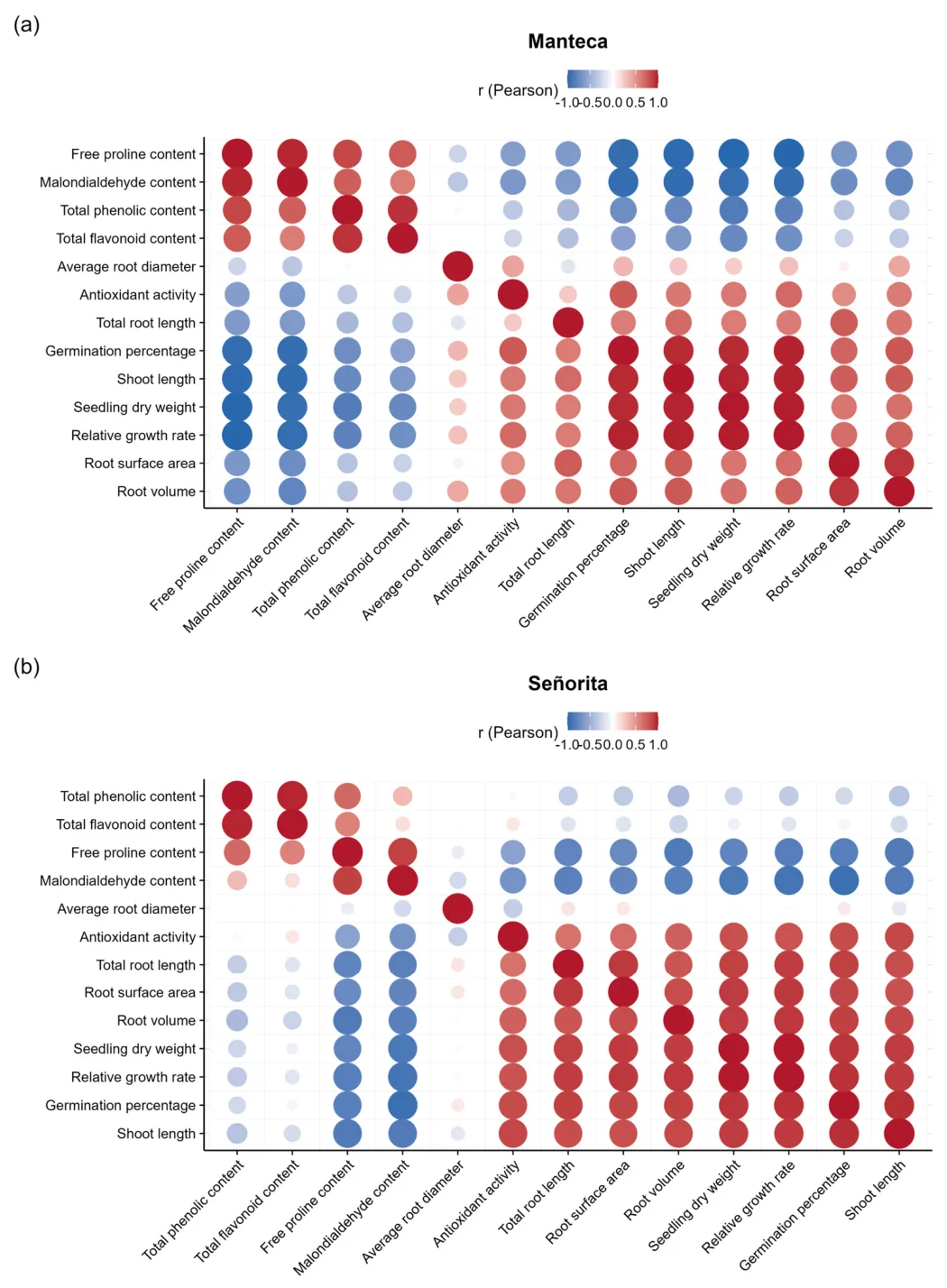
Bivariate correlation structure of growth, morphological and biochemical variables. Correlation matrix, cv. Manteca (**a**) and cv. Señorita (**b**); dot size and color scale with Pearson’s r. Growth and biomass variables are strongly intercorrelated in both cultivars, while average root diameter is essentially uncorrelated with the rest of the trait set.

**Figure 13:**
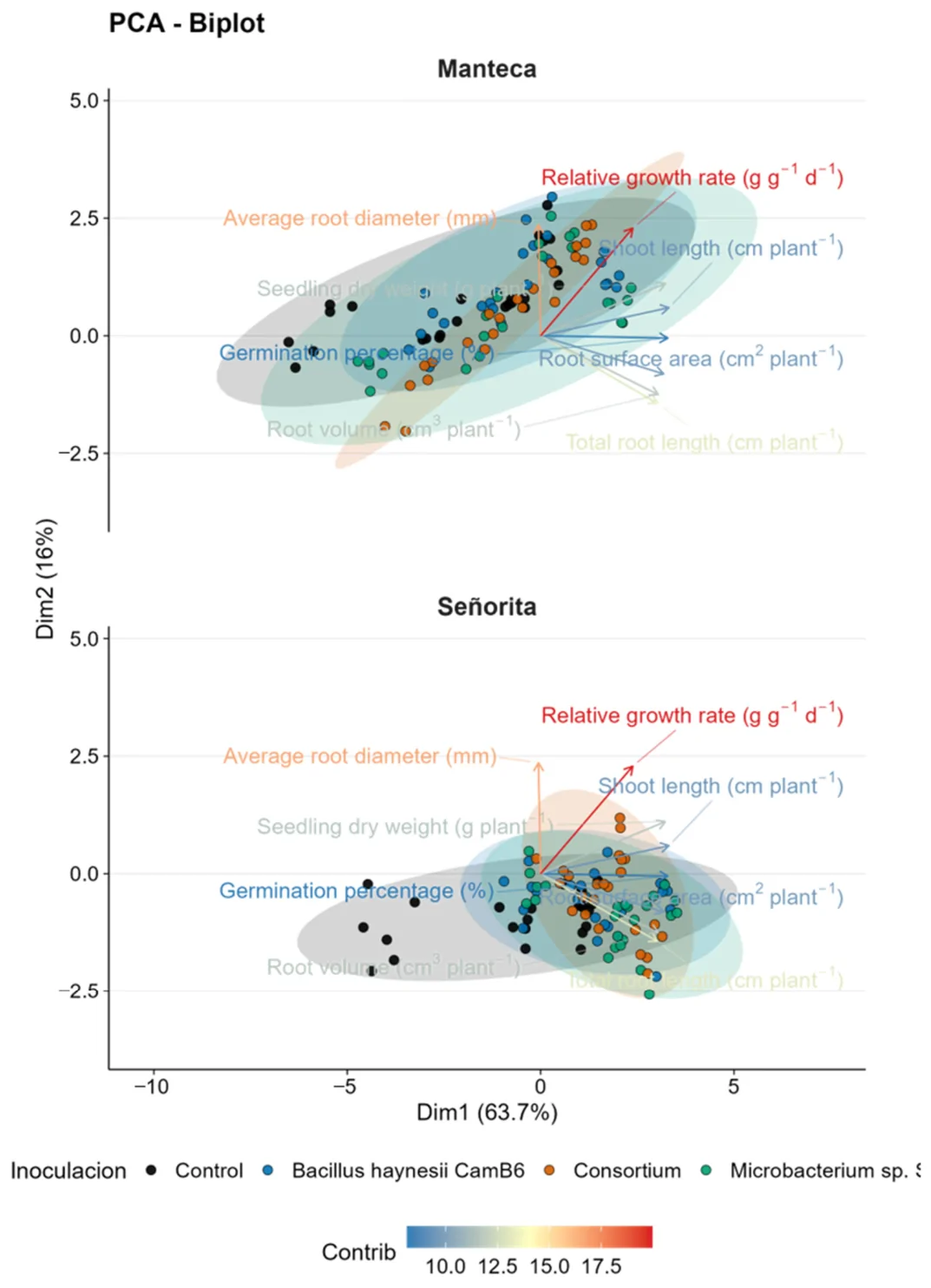
Principal component analysis of the core growth and morphological trait set. Biplot by cultivar (Manteca, (**top**); Señorita, (**bottom**)) and inoculation treatment. PC1 = 63.7%, PC2 = 16.0% of total variance. Points are individual seedlings, coloured by inoculation treatment; the shaded ellipses delimit the corresponding treatment groups, and the arrows are the variable loadings on PC1 and PC2. PC1 separates well-watered, inoculated seedlings from severely stressed, non-inoculated ones; PC2 reflects root diameter and relative growth rate, independent of overall plant size.

**Figure 14:**
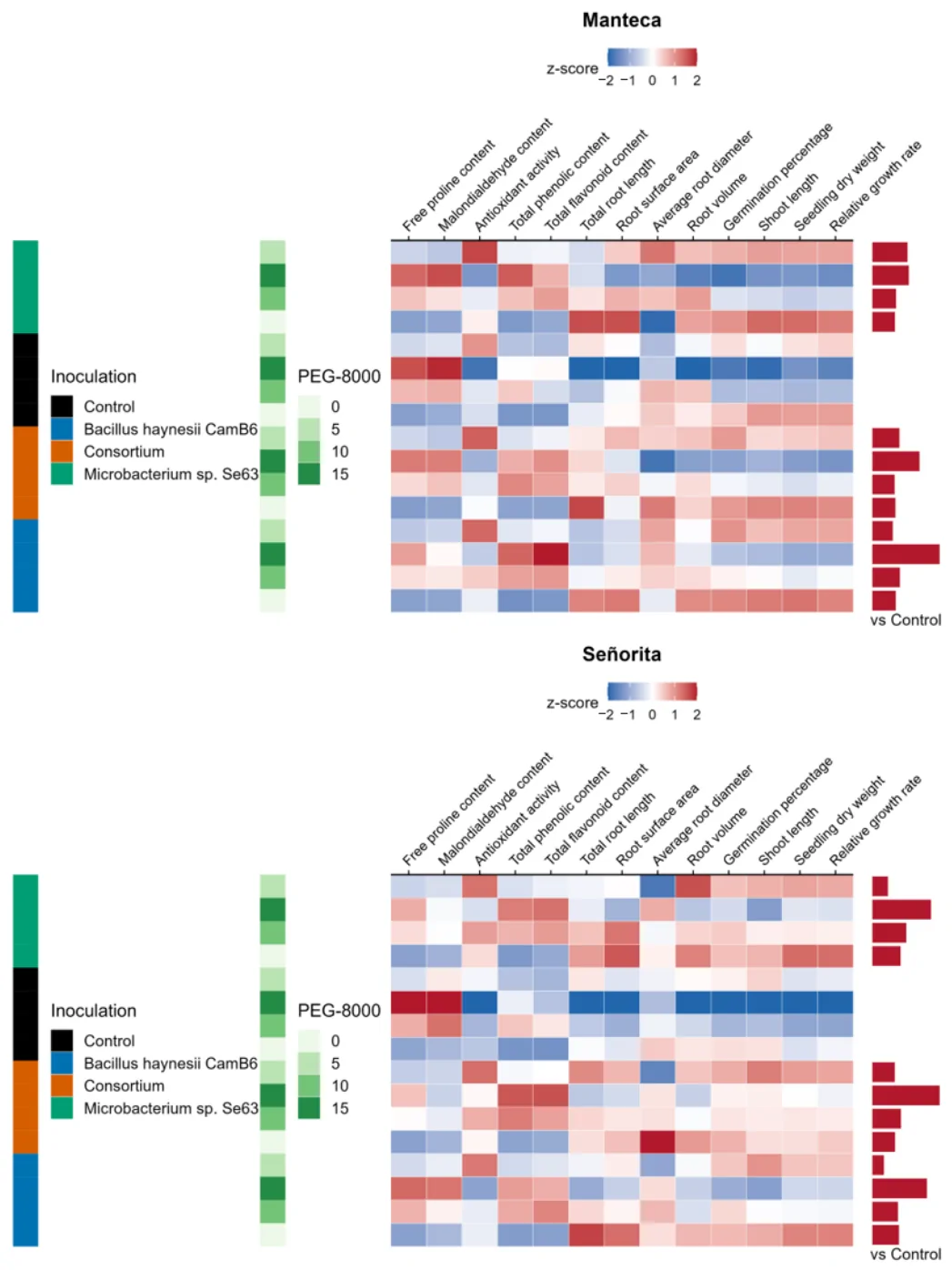
Treatment × variable heatmap of the phenotypic response with hierarchical clustering. Standardized trait values by treatment and PEG-8000 level, cv. Manteca (**top**) and cv. Señorita (**bottom**). Clustering shows a graded, dose-dependent phenotypic trajectory in cv. Manteca, whereas cv. Señorita remains comparatively homogeneous across the stress gradient except when both are severely stressed and non-inoculated.

Growth and biomass variables were strongly intercorrelated (r = 0.59–0.87; Figure 12), with average root diameter the only trait essentially uncorrelated with the rest of the set. A principal component analysis on the core growth and morphological variables resolved 63.7% of variance on PC1 and 16.0% on PC2 (Figure 13). PC1, loading comparably on all variables except average root diameter, separated well-watered, inoculated seedlings from severely stressed, non-inoculated ones; PC2 was dominated by root diameter and relative growth rate, an axis orthogonal to overall plant size. A permutational multivariate analysis of variance confirmed PEG level as the dominant driver of phenotypic dispersion (R^2^ = 0.386, *p* = 0.001), followed by cultivar (R^2^ = 0.229) and inoculation (R^2^ = 0.092), with all interactions also significant but of smaller effect size. Hierarchical clustering showed a graded, dose-dependent phenotypic trajectory in ‘Manteca’, whereas ‘Señorita’ remained comparatively homogeneous across the stress gradient except when both severely stressed and non-inoculated (Figure 14).

Contrasting the consortium mean against the average of its two single-strain components across all Cultivar × PEG combinations (19 of 104 contrasts were significant after Benjamini–Hochberg correction) showed a stress-conditional pattern: under non-stressed conditions, the consortium underperformed the single-strain average for several growth and root traits in both cultivars. Under moderate-to-severe stress, this reversed, with the consortium significantly exceeding the single-strain average for root length in ’Manteca’ (PEG 5–15%) and for shoot length and germination percentage in ’Señorita’ at PEG 15%.

## 3. Discussion

To our knowledge, this is the first study to combine a Chilean thermophilic isolate (*Bacillus haynesii* from an Andean hot spring) with a Chilean psychrotolerant isolate (*Microbacterium* sp. from Antarctic soil) as a consortium on *P. vulgaris* under PEG-induced drought, and the first to explicitly resolve the conditionality of the consortium benefit on stress severity across cultivars of contrasting intrinsic tolerance. The dataset therefore supports a deliberate design principle: bioinoculant consortia should match the abiotic profile of their target agro-ecology rather than rely on universal mixtures.

### 3.1. Screening of Plant-Growth-Promoting Traits

The recruitment of extremophile-origin bacteria as PGPB is an emerging strategy in its own right: bacteria isolated from arid and environmentally extreme sites, including biofilm-forming rhizobacteria from the dry East Azerbaijan grasslands of Iran [11] and *Bacillus* spp. from the Atacama Desert rhizosphere in Chile [12], have been shown to transfer stress-relevant functional traits to crop hosts under controlled water deficit, supporting the premise that bacterial lineages adapted to physicochemical extremes carry PGPB functions of agronomic interest. The qualitative in vitro screen (Table 1) revealed a complementary, largely non-overlapping trait profile: Se63 was positive for phosphate solubilization, siderophore production, and lipase activity, all absent in CamB6, whereas CamB6 was positive for catalase, protease, and HCN production, all absent in Se63; both strains expressed ammonia production, a mucoid phenotype, and apparent N_2_ fixation, with comparable amylase activity. This trait partitioning is consistent with a division-of-labor model of consortium function, in which the net benefit of combining strains depends on whether their complementary traits outweigh the metabolic cost of their functional redundancy under a given stress level. Comparable partitioning has been reported as the substrate of consortium benefit in drought-stressed maize [16] and, with an explicit split of function between partner strains, in a two-strain alfalfa consortium in which one strain drove aboveground and photosynthetic gains and the other drove root growth and oxidative protection under drought [17]; the principle aligns with broader reviews of PGPR-mediated drought alleviation [3,4].

### 3.2. Phosphate Solubilization

The pattern of phosphate mobilization across the four P sources reinforces this complementarity and adds a layer of substrate specificity (Figure 1). Se63 alone released more soluble P than the consortium from tricalcium, aluminum, and iron phosphate, with the largest difference on tricalcium phosphate, whereas sodium phytate mobilization was uniformly low and indistinguishable between treatments. That adding CamB6 did not improve, and in most inorganic sources, reduced solubilization relative to Se63 alone indicates that, for P mobilization specifically, the two strains compete rather than cooperate. Source-dependent outcomes of this kind are consistent with the broader phosphate-solubilizing-bacteria literature, where the identity of the P source shapes strain-level solubilization efficiency [19], and active engineering of phosphate-solubilizing bacterial communities to exploit or avoid such interactions is itself an area of current methodological development [20], underscoring that consortium-assembly rules for P mobilization remain only partly predictable from single-strain traits. Whether the specific strain combination behaves additively or competitively is not fixed: a recent field trial combining two phosphate-solubilizing strains with distinct, P-source-modulated organic-acid profiles achieved complementary rather than competitive agronomic performance in soybean [18], indicating that this outcome is a property of the specific strain pair and P source rather than a universal rule of mixing bacteria.

The apparent discrepancy between the in vitro P-solubilization ranking (Se63 > consortium) and the *in planta* ranking under water deficit (consortium > single strains in ‘Señorita’) is expected rather than contradictory, for three reasons. First, plate- and broth-based P solubilization is a weak predictor of plant growth promotion. In a collection of phosphate-solubilizing bacteria native to acid soils, the capacity to solubilize P in vitro was explicitly found not to be associated with the ability to promote *P. vulgaris* growth [21], and a critical assessment of phosphate-solubilizing microorganisms concluded that the quantities of P mobilized under controlled conditions are insufficient to alter the crop’s nutritional environment, with plant benefit arising instead over longer time scales through turnover of the microbial biomass [22]. Second, where phosphate-solubilizing rhizobacteria do promote growth, the effect is frequently mediated by root-trait modification rather than by rhizosphere P release: in wheat, isolates with the lowest solubilizing capacity produced some of the largest gains in root phosphatase activity and shoot P, and root and shoot physiology responded more strongly than rhizosphere P solubilization [23]. That pattern matches our data, because the consortium was not the best P solubilizer but was the best root colonizer at every PEG level in both cultivars (Figure 5B) and produced the largest root-architecture and biomass gains in ‘Señorita’ under severe stress. Third, and decisively for this system, the germination and early-vigor assay was conducted on a sterile cotton substrate moistened only with PEG-8000 solution, without added phosphate or mineral nutrients (Section 4.7), so early seedling growth was supported by seed reserves. P mobilization therefore cannot be the mechanism underlying the consortium’s in plant advantage in this experiment, and the two assays are not measuring the same function: the NBRIP assay reports a nutritional trait expressed in a P-limited, osmotically benign medium, whereas the seedling assay reports osmotic tolerance, root colonization, and hormonal modification under water deficit.

### 3.3. Indole-3-Acetic Acid Production

IAA production was numerically highest for CamB6, intermediate for the consortium, and lowest for Se63, but the omnibus test fell short of significance at this sample size (one-way ANOVA, *p* = 0.060, η^2^ = 0.609); even the largest pairwise contrast (CamB6 vs. Se63) showed a large effect size (Hedges’ g = 2.03) that did not survive Tukey correction. Three points should be read together. First, a large effect size paired with a non-significant pairwise contrast is consistent with a real biological difference detectable only with greater replication. Second, the in vitro rank order CamB6 > consortium > Se63 is the opposite of the in vivo growth rank observed in ’Señorita’ under PEG 15% (consortium > CamB6 ≈ Se63), indicating that in vitro IAA output alone did not determine seedling performance. Third, IAA at these magnitudes falls within the range associated with PGPR-mediated root and biomass enhancement in other systems [24,25], including a recent report in which IAA-producing *Bacillus megaterium* isolates screened under combined PEG and NaCl-induced osmotic stress, applied singly and as a consortium, enhanced root architecture and reduced malondialdehyde accumulation in drought-stressed sugarcane [26], consistent with the broader drought-alleviation framework [2,3,4]. This apparent paradox— that the most prolific in vitro producer is not the top in vivo rescuer in every cultivar—is reconciled by recognizing that CamB6’s contribution to root architecture is expressed only when the strain retains a functional presence in the rhizosphere under stress, which our colonization data show occurred in ’Manteca’ but not in ’Señorita’ at PEG 15% (Figure 5B).

### 3.4. Bacterial Tolerance to PEG-8000-Induced Osmotic Stress

The in vitro PEG-8000 tolerance assay defines the physiological envelope within which the in vivo effects must be interpreted. CamB6 maintained measurable turbidity across the entire gradient up to 25% *w*/*v*, with a sharp decline only above 20%, and the consortium followed a similar profile. This degree of intrinsic osmotic tolerance is comparable to PEG-8000-tolerant PGPR isolated from rice under water scarcity, where active bacterial metabolism under osmotic challenge was itself a precondition for later seed-stage rescue [27], and to PEG-6000-based screens of osmotolerant endophytes in a legume host, where the resulting consortium raised peanut (*Arachis hypogaea*) germination by 91% under water deficit [28].

### 3.5. Strain Compatibility, Thermal Overlap and Co-Culture Behavior

Because the consortium pairs a thermotolerant with a psychrotolerant isolate, its assembly requires explicit justification on three grounds: a shared permissive temperature, absence of mutual inhibition, and stability of the mixed culture.

The thermal niches of the two strains overlap rather than being disjoint. CamB6 grows between 20 and 60 °C and Se63 between 10 and 35 °C (Table S2), so the 27 °C used throughout this study lies within the permissive range of both and near the center of the range of Se63. CamB6 is thermotolerant rather than obligately thermophilic, which is what makes the pairing feasible at a single temperature; an obligate thermophile could not have been combined with Se63 at any common incubation temperature. Temperature nevertheless remains a legitimate concern for consortium composition, because the relative abundance of members of defined bacterial communities shifts substantially with small temperature changes, influenced though not completely dictated by differences in the growth rates of the individual members [29]. For this reason, the consortium was standardized on total viable load rather than on volume, with the two suspensions adjusted separately and mixed in equal CFU proportions immediately before use (Section 4.5 and Section 4.7), so that any divergence in growth rate during preculture could not translate into an unequal starting ratio. Growth of each strain and of the CamB6 + Se63 co-culture in the common semi-synthetic medium and in the two strain-specific maintenance media was followed hourly over 35–37 h (Figure S5). All three cultures grew in the shared semi-synthetic medium at 27 °C, which is the operational requirement for co-cultivation, but their kinetics differed. Se63 entered exponential growth from about 5 h and plateaued near OD_600_ 0.62 by 28 h, whereas CamB6 showed a longer lag, rose steeply between 15 and 28 h, and plateaued at a higher final density near OD_600_ 0.80: Se63 established faster, and CamB6 reached the greater final biomass. This is why the inoculum was equalized on CFU rather than on culture volume or optical density. The co-culture followed a regular sigmoidal trajectory and reached a stable stationary phase near OD_600_ 0.60 by 29 h, and notably it did so best in the semi-synthetic medium, the medium in which every comparative assay and every inoculum preparation was carried out, while growing more slowly in Jensen medium and reaching only about OD_600_ 0.36 in ISP2. The choice of the common medium is therefore not merely a convenience for comparison: it is the condition under which the two strains co-exist best.

We did not perform a formal dual-culture antagonism assay before assembling the consortium, and we state this explicitly rather than infer compatibility retrospectively. Three independent lines of evidence nonetheless indicate that the two strains are not mutually inhibitory under the conditions used here. First, the co-culture in the common semi-synthetic medium described a regular sigmoidal growth curve and reached a stable stationary phase (Figure S5c), and it grew across the entire PEG-8000 gradient with turbidity comparable to that of the monocultures (Figure 3); a strongly antagonistic pair would instead be expected to show a suppressed, truncated, or biphasic co-culture trajectory. Second, both colony morphotypes pigmented for CamB6 and cream/white for Se63 were recovered from root washes of consortium-inoculated plants (Figure 5A), showing that neither partner excluded the other during root colonization. Third, the consortium produced the highest, or joint-highest, culturable count at every PEG level in both cultivars (Figure 5B), which is difficult to reconcile with strong reciprocal inhibition.

On the specific question of antibacterial compounds, Se63.02b was originally characterized in a genomic and metabolomic screen of Antarctic bacteria in which it displayed antibacterial activity and in which antibiotic biosynthetic gene clusters were identified [15]. The activity reported for this strain was directed against Gram-negative targets and was dependent on culture medium composition; activity against *Bacillus* spp. was not evaluated in that study, and we did not test it here. For CamB6, the described secondary metabolism concerns melanin pigment, exopolysaccharide, and biofilm production under stress rather than antibiosis [13,14]. This remains an acknowledged gap rather than a resolved question, and it is the reason we refrain from claiming general compatibility between the two strains.

Two further considerations bear on how much weight in vitro compatibility testing should carry. Compatibility screening clearly matters: co-inoculation of *Azospirillum argentinense* with two different fluorescent pseudomonads produced synergy with one partner and mutual interference with the other in lettuce [30]; compatibility among potato bioinoculants determined whether combined inoculation was synergistic or antagonistic [31]; and relieving antagonism between two *Bacillus velezensis* strains improved biofilm formation, root colonization and the plant benefit of the pair [32]. Systematic, antagonism-free consortium design has since been formalized in double-agar-layer screening frameworks [33], and such an assay is the appropriate next step for this strain pair. At the same time, in vitro antagonism is an imperfect predictor of *in planta* outcome: a *Streptomyces microflavus*–*Trichoderma harzianum* pair showed strong reciprocal antagonism on agar and in liquid co-culture yet exerted a synergistic effect on tomato seedlings greater than either partner alone, leading the authors to question the usefulness of in vitro assays alone for consortium design and to recommend in vivo evaluation [34]. Our data are consistent with that position. The consortium underperformed the single-strain average for several quantitative traits under non-stressed conditions yet exceeded it under moderate-to-severe stress, so the sign of the interaction is set by the assay environment rather than being an intrinsic property of the pair. The co-culture data point to the same conclusion: in the shared medium, the mixed culture plateaued near OD_600_ 0.60; that is, it did not exceed the better-performing monoculture (CamB6, near OD_600_ 0.80) despite receiving the same total viable load, which is what competition for a common substrate under resource-saturated conditions predicts and is consistent with the loss of consortium advantage we observed in planta in the absence of stress. The observation that multi-trait efficiency and interactivity of bacterial consortia are themselves stress-dependent, with co-culture traits such as biofilm formation changing under osmotic challenge, supports the same conclusion [35].

### 3.6. Biochemical Determinations in Germinating Seeds

Total phenolic content and DPPH radical-scavenging activity in germinating seeds showed a transient peak in inoculated seeds at 24 h that reversed by 48–72 h, when non-inoculated seeds surpassed all inoculated treatments in both cultivars (Table S1). This early, transient rise is consistent with a rapid microbial priming effect on seed defensive metabolism during imbibition; PGPB-based seed biopriming more generally has been shown to modify early germination-associated biochemistry and seedling vigor [36], although the specific rise-then-reversal kinetics documented here have not, to our knowledge, been previously reported for this endpoint and are interpreted as a plausible early kick-start effect rather than as a mechanistically established one. The reversal by 48–72 h, rather than a sustained inoculation advantage, suggests that the bacterial contribution to seed-stage antioxidant metabolism in this system is better described as an early, transient effect than as a persistent enhancement a distinction relevant to interpreting the biochemical rescue observed later, at the seedling stage (Section 3.10).

Two mechanistic questions follow from these kinetics: whether early inoculation protects the germinating seed well enough that less endogenous phenolic investment is required later, and whether bacterial colonization suppresses, induces, or otherwise modulates the phenylpropanoid pathway. The available evidence supports modulation with a temporal sign change rather than simple induction or suppression. PGPB are well documented to stimulate phenolic biosynthesis through activation of the phenylpropanoid pathway, with reported increases ranging from 9% to over 200% [37], and the early rise observed here at 24 h is consistent with that literature. When the phenylpropanoid response is examined in relation to growth rather than in isolation, however, the association inverts: across three plant species and three rhizobacterial species, suppression of flavonoid biosynthesis was associated with rhizobacteria-mediated growth promotion, whereas elevated flavonoid levels were associated with growth reduction [38]. Our seed-stage data follow that pattern: inoculated seeds showed the highest radical-scavenging activity at 24 h and the lowest phenolic content from 48 h onwards, while producing greater seedling vigor and are therefore better read as bacterially mediated relief of the oxidative burden than as a loss of defensive capacity.

This interpretation is consistent with the growth–defense trade-off framework. Phenolic biosynthesis draws carbon from primary metabolism, and stress-induced phenolic accumulation is linked to reduced growth through the proline redox cycle and the oxidative pentose phosphate pathway [39]; allocation between growth and defense is coordinated rather than independent, so lowering the stress signal lowers the demand for defensive investment [40]. A recent multi-omics analysis of PGPR-treated plants resolved this as an explicit temporal partition, with an early metabolic priming phase preceding a later phase of proliferative expansion and structural remodeling [41]. Read through that lens, the 24 h peak corresponds to priming and the subsequent decline to the transition into expansion, in which the inoculated seedling reallocates carbon from phenylpropanoid synthesis towards growth. Importantly, the direction reverses again at the seedling stage, where total phenolic and flavonoid contents were increased by inoculation under stress (Figure 11). The modulation is therefore stage-specific, and any general statement that these strains simply induce or suppress phenolic metabolism would be an oversimplification. Direct measurement of phenylalanine ammonia-lyase activity and of *PAL*, *C4H* and *4CL* transcript abundance across the same time course would be required to establish the mechanism; the interpretation above is offered as a hypothesis consistent with the data rather than as a demonstrated pathway effect.

### 3.7. Confocal Laser Scanning Microscopy of Root–Bacteria Association

The confocal and culturable data show that all three bacterial treatments colonized *P. vulgaris* roots: SYTO9 staining revealed dense viable-cell signal in all inoculated treatments regardless of PEG-8000 level, while propidium-iodide signal indicating membrane-compromised cells was most extensive in CamB6 and most limited in the consortium. The CamB6 signature is consistent with the membrane-stability cost of monoculture under stress, but the near-complete loss of recoverable CamB6 from ’Señorita’ roots at PEG 15% shows that rhizosphere competence in vivo cannot be predicted from in vitro tolerance alone. The consortium, in contrast, produced the highest colony count from root washes at every PEG level in both cultivars, indicating more stress-robust root colonization by the mixed culture than by either pure culture; comparable community-level stabilization of rhizosphere populations under drought has been documented using co-occurrence-network approaches [42]. The cultivar-dependent reciprocal ranking of CamB6 and Se63 on root washes, CamB6 outperforming Se63 in ’Manteca’ but underperforming it in ’Señorita’, parallels reports that the same PGPR consortium’s effectiveness in a legume host depends on host genotype as much as on strain identity [43], indicating that genotype–microbe compatibility is a non-trivial constraint when scaling these inoculants beyond the two cultivars tested here.

### 3.8. Germination and Early-Vigor Indices

The germination and early-vigor results confirm that ’Señorita’ is intrinsically more stress-tolerant than ’Manteca’ and identify the inoculation strategy as stress-conditional in a cultivar-specific way: CamB6 rescued ’Manteca’ germination most effectively at moderate stress, while the consortium rescued ’Señorita’ germination, shoot length and dry weight most effectively at the most severe stress level tested. This decoupling a single strain rescuing the more sensitive cultivar under moderate stress, the consortium rescuing the more tolerant cultivar under severe stress is consistent with evidence that *Bacillus velezensis* inoculation, alone and combined with exogenous acetylsalicylic acid, increased yield and relative water content and reduced oxidative-stress markers in field-grown *P. vulgaris* under deficit irrigation (70% of full water requirement; [5]), and with evidence that indigenous, locally adapted rhizobial strains outperform a reference commercial strain in sustaining *P. vulgaris* biomass under severe drought [7] a precedent directly relevant to the rationale for testing extremophile-origin, locally sourced strains here. A concurrent report in the same host species found that *Bacillus subtilis* inoculation alleviated drought-induced reductions in biomass, photosynthetic pigments, and relative water content in *P. vulgaris* while attenuating oxidative damage and raising osmolyte and antioxidant pools, with multivariate analysis confirming the overall benefit of microbial inoculation [6], corroborating the direction, though not the cultivar-specific magnitude, of the rescue documented here. The pattern also aligns with the general framework that PGPR benefit under drought scales with the severity of the baseline stress [2,3,4,27]. In ’Manteca’, CamB6’s rescue reached significance at moderate stress but not at the most severe level tested, suggesting a ceiling to single-strain rescue under severe osmotic stress that the consortium did not hit in ’Señorita’. The restoration of a positive relative growth rate in severely stressed ’Señorita’ seedlings by consortium inoculation, from a negative value indicating net biomass loss in non-inoculated controls, is among the most concrete pieces of evidence in this study for a rescue effect specific to the consortium under severe osmotic deficit.

The dependence of the response to inoculation is supported in the literature by three explanations that are not mutually exclusive. First, host genotype controls responsiveness to bacterial auxin, and this has been demonstrated in *P. vulgaris* itself: the genotypes BAT477 and DOR364 differ in root developmental responsiveness to the auxin-producing rhizobacterium *Azospirillum brasilense* Sp245; the difference tracks the response to bacterially produced auxin rather than to the bacterium as such, and quantitative trait loci for basal-root responsiveness to auxin were mapped in a recombinant inbred population derived from that cross, one of which explained 36% of the phenotypic variation [44]. Because the rescue of root architecture in our experiment tracked the in vitro IAA ranking (Section 3.3), genotypic variation in auxin responsiveness is a plausible primary cause of the Manteca–Señorita asymmetry and is directly testable in this germplasm. Second, cultivars differ in root exudate composition, which governs both colonization and the functional traits the bacteria express: variety-specific exudation determined PGPR colonization and the growth response in soybean [45], and in barley, cultivar genotype shaped microbiota composition, the phenotypes and genotypes of the recruited bacterial populations, and their gene expression through differential exudate secretion [46]. Our colonization data are consistent with an exudate-mediated effect, since CamB6 and Se63 reversed rank on root washes between the two cultivars, with near-complete loss of recoverable CamB6 from ‘Señorita’ at PEG 15% (Figure 5B). Third, the two cultivars differ in intrinsic stress tolerance, which sets the baseline against which any rescue is measured. We did not measure root exudate profiles or auxin-response phenotypes, so these remain candidate mechanisms requiring verification rather than established causes.

Two specific features of the dose–response deserve comment. In ‘Señorita’, the inoculation treatments were statistically indistinguishable at PEG 5% but clearly separated at PEG 15%. This is the expected signature of a stress-conditional benefit acting against a tolerant background: at the milder level, the non-inoculated control of this cultivar remained close to its unstressed performance (94.5% germination at PEG 0, declining to 65.0% only at PEG 15%), leaving little deficit for inoculation to recover, so all treatments converge on the same ceiling and differences cannot be resolved. Only when the non-inoculated control is driven into net biomass loss a negative relative growth rate at PEG 15% does the dynamic range open sufficiently for the treatments to separate, and it is precisely there that the consortium restored a positive growth rate. The same logic explains why the consortium exceeded the single-strain average only under moderate-to-severe stress in the multivariate contrast (Section 3.11): a benefit defined relative to a deficit cannot be detected when the deficit is absent. In ‘Manteca’, by contrast, inoculation effects were smaller and largely did not reach significance across the PEG gradient. Three factors plausibly contribute. This cultivar is intrinsically more susceptible, with germination falling to 31.0% in the non-inoculated control at PEG 15%; when a large fraction of seeds fails to germinate, between-replicate variance rises, and the power to detect a rescue falls, which is consistent with the +23.8% dry-weight gain conferred by CamB6 at PEG 15% failing to reach significance (*p* = 0.116) while a comparable gain at PEG 10% (+34.0%) did. Colonization is also asymmetric between cultivars, and the reciprocal ranking of CamB6 and Se63 means the consortium cannot deploy both partners equally in both hosts. Finally, host responsiveness to bacterial auxin is genotype-dependent in this species [44], so a cultivar with low auxin responsiveness would show a muted root-architecture response even under effective colonization. Distinguishing a genuine absence of effect from insufficient statistical power in ‘Manteca’ requires a dedicated experiment with higher replication at the severe-stress level, which we identify as a priority alongside the field validation already proposed.

### 3.9. Image-Based Analysis of Root System Architecture

Root architecture parallels the shoot-level results: inoculation approximately doubled root volume in stressed ’Manteca’ (with CamB6) and stressed ’Señorita’ (with the consortium), tracking the IAA profile measured in vitro (CamB6 > consortium > Se63; Figure 2) and consistent with the mechanistic model in which auxin-producing strains enhance root elongation under stress [2,3,4,24,25]. Average root diameter was essentially uncorrelated with the other architectural traits, indicating that the inoculation-induced enhancement of root length and surface area occurred through a finer-root system exploring a larger soil volume per unit carbon rather than through thicker roots, a trait consistent with drought avoidance through improved water acquisition. The strain-specific inversion in which strain produced thicker roots under severe stress (CamB6 in ’Manteca’, Se63 in ’Señorita’) reinforces the conclusion that root-architecture responses to bacterization are jointly shaped by inoculum composition and host genotype rather than by strain identity alone.

### 3.10. Biochemical Determinations in Seedling Tissues

Free proline, MDA, and Trolox-equivalent antioxidant capacity in seedling tissue provide the cellular context for the morphological results. Free proline rose steeply across the PEG gradient in non-inoculated controls, consistent with a classical osmotic-adjustment response to water deficit in common bean, with only a modest and cultivar-dependent inoculation effect. MDA accumulation, a marker of lipid peroxidation, was significantly attenuated by CamB6 in ’Señorita’ but not in ’Manteca’ at any PEG level a result that mirrors the cultivar × inoculation asymmetry seen throughout the dataset and aligns mechanistically with evidence that ACC-deaminase-producing PGPR reduce ethylene-mediated lipid peroxidation and lower drought-induced ABA accumulation, thereby improving water-use efficiency, in another woody perennial under drought [47], and more broadly with the framework that antioxidant and ACC-deaminase activities are among the main routes through which PGPR attenuate oxidative damage under abiotic stress [2,3,4]. Trolox-equivalent antioxidant capacity increased only with the consortium in ’Señorita’ at the most severe stress level, with no effect detected in ’Manteca’ at any PEG level, a cultivar specificity that cannot be explained by strain tolerance alone, since both cultures survived the applied osmotic potential in vitro, and that instead indicates a host-genotype-driven component in the antioxidant response to inoculation. Total phenolic and flavonoid contents were elevated by PEG stress and further increased by inoculation under stress, an additive pattern consistent with the seed-stage biochemistry (Section 3.5) and mechanistically plausible through PGPR-induced upregulation of phenylpropanoid-pathway intermediates under abiotic stress [3,4].

### 3.11. Multivariate Structure of the Phenotypic Response

The principal component analysis, PERMANOVA, and hierarchical clustering integrate the trait-level results into a coherent multivariate picture. PC1 separated well-watered, inoculated seedlings from severely stressed, non-inoculated ones, reproducing at the multivariate level the pattern visible in each individual endpoint, while PERMANOVA confirmed PEG level as the dominant driver of phenotypic dispersion, ahead of cultivar and inoculation, consistent with the per-endpoint effect-size partition and with the interpretation that inoculation acts as a modifier of the stress response rather than as a primary driver of phenotype. The single most integrative result is the consortium contrast: across all cultivar × PEG combinations, the consortium significantly differed from the mean of its single-strain components in 19 of 104 contrasts after correction for multiple comparisons, with the direction of the difference flipping with stress severity. Below PEG stress, the consortium underperformed the single-strain average, consistent with competitive displacement between CamB6 and Se63 in a resource-saturated environment; under moderate-to-severe stress, this reversed, consistent with a model in which division of labor between strains becomes net-beneficial only once stress is high enough that each strain’s redundant metabolic functions cost more than they save. A stress-conditional consortium advantage of this kind, absent or negative under benign conditions, positive under water deficit, has been documented directly in maize [16] and, with an explicit functional division of labor between partner strains, in a droughted legume consortium [17]. A closely analogous pattern was reported for grapevine, where, among four tailored rhizobacterial consortia, one improved shoot growth mainly under well-watered conditions while a second conferred its benefit specifically under water deficit, maintaining photosynthesis and stomatal conductance when all other treatments showed near-complete stomatal closure [48], providing direct confirmation that different consortia within the same functional-trait framework can partition their benefit across the stress gradient exactly as CamB6 + Se63 did here. The overall pattern is conceptually consistent with community-level resilience patterns in drought-stressed rhizosphere microbiomes more broadly [42,49].

### 3.12. Limitations of the PEG-Based Approach

These results should be interpreted within the limits of PEG-based simulation of water deficit. PEG-8000 imposes an osmotic dehydration that does not reproduce the temporal dynamics, the soil-matric component, or the root-zone heterogeneity of progressive soil drying [50], and PEG exposure has been shown to alter root anatomy in barley, which constrains the extrapolation of image-based root-architecture responses to field water deficit [51]. The stress-conditional consortium benefit reported here therefore requires confirmation in a representative Andisol under naturally fluctuating water status before it is operationalized as a deployment rule.

## 4. Materials and Methods

### 4.1. Bacterial Strains and Culture Conditions

Two previously characterized extremophile bacterial strains of contrasting thermal origins were used: *Bacillus haynesii* CamB6 and *Microbacterium* sp. Se63.02b (hereafter Se63)*. B. haynesii* CamB6 was isolated from the Campanario hydrothermal spring (Andes Mountains, Maule Region, Chile) and identified through sequencing of the 16S ribosomal RNA gene (GenBank MZ298610; Marín-Sanhueza et al. [14]). It was selected because of its origin in a thermal spring and its documented ability to form biofilms, films, and exopolysaccharides, as well as its production of pigments in response to stress [13]. *Microbacterium* sp. Se63.02b was isolated from Antarctic soil and characterized as part of a genomic and metabolomic bioprospecting study of Antarctic bacteria (GenBank genome accession number: CP050453; Núñez-Montero et al. [15]), and was selected as a cold-adapted representative of a genus that includes plant-associated strains with ACC deaminase activity, auxin and siderophore production, and phosphate solubilization [8]; these characteristics at the genus level provide the functional rationale for the strain, but they were not evaluated in relation to other aspects attributed to it here.

The strains were routinely maintained on Jensen medium (HIMEDIA) for CamB6 and on Yeast Malt Agar (ISP2) medium (HIMEDIA), respectively; colony morphology and culture characteristics of both strains are summarized in Table S2. Because the two strains were originally grown on nutritionally distinct media, a common semi-synthetic medium was formulated to allow direct comparison among monocultures and the CamB6 + Se63 consortium. Nitrogen-source screening used a nitrogen-free basal medium (glucose 10 g L^−1^, K_2_HPO_4_ 2 g L^−1^, KH_2_PO_4_ 1 g L^−1^, MgSO_4_·7H_2_O 0.2 g L^−1^, NaCl 0.5 g L^−1^) supplemented individually with seven nitrogen sources at 1% (*w*/*v*): potassium nitrate, ammonium nitrate, ammonium sulfate, sodium nitrate, sodium nitrite, L-methionine and casein. In parallel, carbon-source utilization was tested in a carbon-free mineral medium (K_2_HPO_4_ 7 g L^−1^, KH_2_PO_4_ 2 g L^−1^, MgSO_4_·7H_2_O 0.1 g L^−1^, ammonium nitrate 0.1 g L^−1^, yeast extract 0.5 g L^−1^) with glucose, sodium acetate, sucrose, maltose, mannitol, or citric acid at 1% (*w*/*v*). Growth was monitored as OD_600_ in liquid and solid media. Based on these assays, a semi-synthetic co-culture medium was formulated containing glucose 5 g L^−1^, (NH_4_)_2_SO_4_ 1 g L^−1^, yeast extract 0.5 g L^−1^, K_2_HPO_4_ 2 g L^−1^, MgSO_4_·7H_2_O 0.2 g L^−1^, and CaCl_2_ 0.1 g L^−1^; for solid medium, agar was added at 12 g L^−1^. This medium supported growth of CamB6, Se63, and their consortium and was used for all subsequent comparative assays. Unless otherwise stated, cultures were incubated at 27 °C and 150 rpm. Inocula were prepared from logarithmic-phase cultures, centrifuged, washed twice in sterile 0.85% (*w*/*v*) NaCl, and adjusted to an OD_600_ of 0.8, corresponding to approximately 1 × 10^8^ CFU mL^−1^.

The thermal growth ranges of the two strains overlap: CamB6 grows at 20–60 °C and Se63 at 10–35 °C, and colony morphology and culture characteristics are summarized in Table S2 and illustrated for pure cultures of both strains in Figure S4. All comparative assays and all plant experiments were therefore conducted at 27 °C, which lies within the permissive range of both strains. Growth of the two strains, and of the CamB6 + Se63 co-culture, in the common semi-synthetic medium and in the two maintenance media (Jensen and ISP2) was followed as OD_600_ hourly for 35–37 h at 27 °C and 150 rpm, with three biological replicates, and is reported in Figure S5; these time courses establish that both strains and the mixed culture grow in the shared medium at the common incubation temperature and quantify the difference in their growth kinetics. No formal dual-culture antagonism assay (cross-streak, double-agar-layer, or well-diffusion) was performed before the consortium was assembled; the compatibility evidence available for this strain pair is therefore indirect and is discussed in Section 3.5.

### 4.2. Screening of Plant-Growth-Promoting Traits

Qualitative screening of plant-growth-promoting (PGP) traits was conducted following the method of Gupta and Pandey [52], with some modifications; all experiments were incubated at 27 °C for 24 h at 150 rpm on an orbital shaker (Brand) with three biological replicates. Ammonia production was analyzed in peptone water (peptone—10 g/L; NaCl—5 g/L); 100 μL of Nessler’s reagent was added to 3 mL of bacterial culture (in the early stationary phase) to observe the color change in the medium (positive: from yellow to brown). To verify hydrogen cyanide production on King’s B agar containing 4.4 g L^−1^ of glycine, Whatman filter paper saturated with alkaline citrate buffer was placed on the lids of Petri dishes (positive: from yellow to orange/brown) and incubated for 4 days at 27 °C. The production of siderophores was assessed on Chrome Azurol S agar for 4 days at 27 °C (orange to yellow halo: positive).

### 4.3. Phosphate Solubilization

The ability of bacterial strains to solubilize inorganic phosphate was first screened qualitatively on solid National Botanical Research Institute’s phosphate growth medium (NBRIP) containing tricalcium phosphate (TCP) as the insoluble P source [53]. The NBRIP medium contained, per liter: glucose 10 g, (NH_4_)_2_SO_4_ 0.1 g, KCl 0.2 g, MgSO_4_·7H_2_O 0.25 g, MgCl_2_·6H_2_O 5 g, and Ca_3_(PO_4_)_2_ 5 g. For the solid medium, agar was added at 15 g L^−1^. Bacterial suspensions were inoculated onto NBRIP agar plates and incubated at 27 °C. The appearance of clear halos around bacterial colonies was considered indicative of TCP solubilization. Four biological replicates were used per strain.

Quantitative phosphate solubilization was determined in liquid NBRIP medium containing TCP. Briefly, 20 mL of liquid NBRIP medium was inoculated with 100 µL of a bacterial suspension obtained from a 24 h preculture in semi-synthetic medium and adjusted to a final concentration of 1 × 10^6^ CFU mL^−1^. Cultures were incubated at 27 °C in the dark under orbital shaking at 150 rpm. After 7 days of incubation, cultures were centrifuged, and the supernatants were filtered through 0.22 µm membranes prior to soluble P determination. Soluble phosphate was quantified colorimetrically using the vanadomolybdophosphoric acid method [54,55], with absorbance measured at 466 nm against a KH_2_PO_4_ calibration curve [55]. The concentration of soluble P was expressed as mg L^−1^. Culture pH was recorded at the beginning of the assay and after 7 days of incubation to evaluate medium acidification associated with phosphate solubilization. Quantitative assays were performed with six biological replicates per strain.

Substrate-dependent P mobilization was additionally assessed using NBRIP liquid medium supplemented with 5 kg m^−3^ of tricalcium phosphate [Ca_3_(PO_4_)_2_] as a P source. Aluminum phosphate (AlPO_4_) and iron phosphate (FePO_4_) solubilization were determined by replacing Ca_3_(PO_4_)_2_ with 4 kg m^−3^ of AlPO_4_ and 6 kg m^−3^ of FePO_4_. The initial pH was adjusted to 5.8 ± 0.1 to approximate the acidic conditions commonly found in volcanic Andisols. The tubes containing 9.9 × 10^−6^ m^3^ of NBRIP medium were inoculated with 1 × 10^−7^ m^3^ of bacterial suspension (10^8^ CFU mL^−1^) and were incubated at 27 °C in the dark under orbital shaking at 150 rpm force for 7 days. After the incubation period, the culture was centrifuged at 6000 rpm for 15 min to remove the cell biomass [55].

### 4.4. Indole-3-Acetic Acid Production

The production of indole compounds was quantified using the Salkowski method [56]. The strains were cultured in a semisynthetic medium containing 5 mM L-tryptophan for 7 days at 27 °C and 150 rpm. The cell-free supernatants were mixed with Salkowski’s reagent (0.5 M FeCl_3_ + 70% perchloric acid). The development of a red color (indicating the formation of indole compounds) upon addition of the Salkowski reagent to the cell-free culture supernatant (4:1) was measured using a UV-vis spectrophotometer at 530 nm, and the absorbance was converted to IAA equivalents using a calibration curve of pure IAA (0–100 µg mL^−1^) [52]. Uninoculated medium, with and without L-tryptophan, served as a blank to correct for the indole background from the yeast extract.

### 4.5. Bacterial Tolerance to PEG-8000-Induced Osmotic Stress

The growth of CamB6, Se63, and the consortium was evaluated in a semi-synthetic medium containing PEG-8000 at concentrations of 0, 5, 10, 15, 20, and 25% (*w*/*v*). The total bacterial load was standardized to 1 × 10^7^ CFU mL^−1^ for monocultures and 0.5 × 10^7^ + 0.5 × 10^7^ CFU mL^−1^ for the consortium. Cultures were inoculated at 1% (*v*/*v*) in 50 mL flasks (20 mL) and incubated at 27 °C and 150 rpm for 24 h (three biological replicates). Because PEG increases viscosity and skews turbidimetric measurements at high concentrations, growth was quantified both as OD_600_ (PEG-matched blanks) and as viable counts (CFU mL^−1^). Osmotic potential per PEG concentration was estimated using Michel [57]; this approximation is acknowledged, as the original Michel and Kaufmann [58] was derived for PEG-6000.

### 4.6. Plant Material and Seed Surface Sterilization

Seeds of *Phaseolus vulgaris* L. cultivars Manteca (Phv18) and Señorita (Phv8) were used to evaluate the effect of bacterial inoculation under PEG-induced osmotic stress. Uniform, healthy seeds were selected manually, rinsed in distilled water, surface-sterilized in 2% (*v*/*v*) sodium hypochlorite for 3 min, and rinsed three times in sterile distilled water. Sterilization efficacy was verified by plating an aliquot of the final rinse on nutrient agar.

### 4.7. Seed Bacterization and Germination Under PEG-8000-Induced Osmotic Stress

Seed bacterization was performed according to Meza et al. [59], with modifications, and germination was assessed under a cultivar × inoculation × PEG concentration factorial structure. Four inoculation treatments were applied to each cultivar: uninoculated control, *B. haynesii* (CamB6), *Microbacterium* sp. (Se63), and the CamB6 + Se63 consortium. Suspensions were prepared from logarithmic-phase cultures and standardized to a total of 1 × 10^8^ CFU mL^−1^ (consortium: 0.5 × 10^8^ + 0.5 × 10^8^ CFU mL^−1^, mixed immediately before use to equalize total load). Surface-sterilized seeds were immersed in a 10:1 (*v*/*w*) suspension-to-seed ratio and agitated gently for 30 min; control seeds were treated identically with sterile semi-synthetic medium. Inoculum density was confirmed by plate counting, and seeds were blotted on sterile filter paper.

Bacterized and control seeds of both cultivars were then placed on a sterile cotton substrate inside 2 L sterile glass containers moistened with sterile PEG-8000 solutions at 0% (no-stress control), 5%, 10%, or 15% (*w*/*v*), corresponding to osmotic potentials of −0.51, −1.32, and −3.21 MPa for 5, 10, and 15% (*w*/*v*), respectively, estimated using the Michel and Kaufmann equation [58]. Containers were covered with a breathable polyurethane membrane and held in darkness at 22 °C for 4 days. Germinated seedlings were transferred to a growth chamber (25 °C, 16 h light/8 h dark) and evaluated 12 days after inoculation (≈ 8 days after germination; approximately BBCH stage 12). Total seedling length, first-leaf length, and root-system traits (Section 4.11) were recorded, with six independent biological replicates per treatment.

For the consortium, the two suspensions were prepared separately, adjusted individually to 1 × 10^8^ CFU mL^−1^, and mixed in equal volumes immediately before seed immersion, giving 0.5 × 10^8^ + 0.5 × 10^8^ CFU mL^−1^ and a total viable load identical to that of the monoculture treatments; standardizing on total load rather than on volume prevents any difference in preculture growth rate between the two strains from producing an unequal starting ratio. Inoculum density was confirmed by plate counting on the day of use. Three seeds were sown per container. After the 4-day dark imbibition period, containers were transferred to a growth chamber set to 25 °C, a 16 h light/8 h dark photoperiod, a light intensity of 340 µmol m^−2^ s^−1^, and 50% relative humidity. The PEG-8000 solutions were not renewed during the assay, so the imposed osmotic potential was established at the start and not readjusted. The presence of both partners on roots of consortium-inoculated plants was verified by the pigmented (CamB6) and cream/white (Se63) colony morphotypes recovered on solid semi-synthetic medium (Figure 5A); colony counts are reported as totals per treatment (Figure 5B) and were not partitioned between partners.

### 4.8. Biochemical Determinations in Germinating Seeds

Germinating seeds of each treatment were sampled at 24, 48, and 72 h after inoculation under each PEG-8000 treatment; sampling was destructive, with separate seed batches for each time point (10 seeds per treatment; three biological replicates). The seeds were washed twice with distilled water, rapidly frozen in liquid nitrogen, and ground; approximately 0.5 g of the resulting tissue was extracted and left overnight in 5 mL of 80% (*v*/*v*) methanol at 4 °C, then centrifuged (10,000 *g*, 15 min, 4 °C) according to Pagano et al. [60]. The supernatant was used for both determinations described below.

*Total phenolic content*. The total phenolic content was quantified using the Folin–Ciocalteu method [61] with modifications [62,63]. In summary, 250 µL of supernatant was mixed with 1 mL of Folin–Ciocalteu reagent (1:10 *v*/*v* in distilled water) and vortexed for 3 min; then 2 mL of 7.5% (*w*/*v*) Na_2_CO_3_ was added, and the reaction was carried out in the dark for 30 min at room temperature. The absorbance was read at 760 nm against a reagent blank. The total phenolic content was expressed as mg of gallic acid equivalents (GAE) per g of fresh weight against a gallic acid curve (0, 10, 20, 50, 100 mg L^−1^; r^2^ > 0.99) [64,65].

*DPPH radical-scavenging activity*. Using the same extract, DPPH radical-scavenging activity was measured following Brand-Williams et al. [66] with modifications [67,68]. A 100 µL aliquot of extract was mixed with 3.9 mL of 0.06 mM methanolic DPPH, vortexed, and incubated in darkness for 30 min at 25 °C. Absorbance was read at 517 nm against a methanol blank. Radical-scavenging activity was calculated according to Equation (1).

(1) $$\left(\%\right)RSA=\frac{\;1-A_{\mathrm{sample}}}{A_{\mathrm{control}}}\times 100$$

where *A*_sample_ is the absorbance of the reaction mixture containing the extract and *A*_control_ is the absorbance of the DPPH solution without extract. Ascorbic acid (0–2 mM) was included as a positive control to verify assay performance [60].

### 4.9. Confocal Laser Scanning Microscopy of Root–Bacteria Association

Root–bacteria association was assessed by confocal laser scanning microscopy after seed bacterization and early seedling growth. Roots from inoculated seedlings and from non-inoculated controls were sampled at 7 days after inoculation; segments of approximately 1 cm were excised from the primary root and/or the lateral-root zone, rinsed gently in sterile phosphate-buffered saline to remove loosely attached cells, and processed immediately for fluorescence staining.

Tissue-associated bacterial cells were stained with the LIVE/DEAD BacLight bacterial viability kit (SYTO 9 and propidium iodide; Thermo Fisher Scientific, Waltham, MA, USA) following the manufacturer’s instructions with minor modifications. Briefly, root segments were incubated with the staining solution for 15 min in darkness at room temperature, rinsed gently to remove excess dye, and mounted in sterile buffer on glass slides. Non-inoculated roots stained in parallel served as controls for root autofluorescence and non-specific staining.

Samples were examined on a Leica Stellaris 5 microscope (Leica Microsystems, Wetzlar, Germany) at 60× magnification, with SYTO 9 and propidium iodide detected at excitation/emission settings of 485/498 nm and 535/617 nm, respectively. Fluorescence channels were acquired separately and merged. Green fluorescence was interpreted as bacterial cells with intact membranes and red fluorescence as cells with compromised membranes and/or extracellular DNA associated with bacterial aggregates or biofilm-like structures; because propidium iodide also labels plant nuclei and DNA, the red signal was interpreted with caution and not attributed exclusively to bacteria. Root–bacteria association was assessed qualitatively from representative images obtained from three independent biological replicates per treatment and two root segments per replicate.

As a complementary culture-dependent assessment, culturable root-associated bacteria were quantified 7 days after inoculation. A separate central root segment was aseptically excised, transferred to 2 mL of sterile distilled water, and vortexed to dislodge associated bacterial cells [69]. The resulting suspension was serially diluted to 10^−5^, and 100 µL of each appropriate dilution was spread onto solid semi-synthetic medium [70]. Following incubation at 27 °C [71], colonies were counted and expressed as CFU per root segment after correction for the dilution factor and plated volume. Non-inoculated roots were processed identically as negative controls. CamB6 and Se63 colonies were presumptively differentiated according to their characteristic orange salmon and cream white morphologies, respectively, using pure-culture reference plates for comparison [72].

### 4.10. Germination and Early-Vigor Indices

Germination was recorded daily; a seed was scored as germinated at radicle protrusion ≥ 2 mm. Final germination percentage and mean germination time were calculated, and early-vigor indices were calculated following Sarkar et al. [73]. Equations (2)–(5): relative germination, toxicity percentage, tolerance index and seedling vigor index, respectively.

(2) $$Relative\;germination\;(\%)=\frac{\;germination\;seeds\;in\;treatment}{\;germination\;seeds\;in\;control\;}\times 100$$

(3) $$Toxicity\;\left(\%\right)=\frac{radicle\;length\;\left(control\right)-radicle\;length\;\left(treatment\right)}{radicle\;length\;\left(control\right)}\;\;\times \;\;100$$

(4) $$Tolerance\;index=\frac{Mean\;length\;of\;longest\;root\;in\;treatment\;}{Mean\;length\;of\;longest\;root\;in\;control}$$

(5) Seedling vigor index=[(mean root length+mean shoot length)]×% of germination

For all indices, the control was the treatment without inoculation at the same PEG level/the stress-free condition (0% PEG). Since relative germination can reach saturation under mild osmotic stress, the average germination time is reported along with Equations (2)–(5) to preserve the dynamic range across all PEG levels.

Seedling relative growth rate was calculated as the rate of biomass accumulation per unit biomass per unit time. Equation (6)

(6) $$RGR\;\left({\mathrm{gg}}^{-1}d^{-1}\right)=\frac{\ln W_{2}-\ln W_{1}}{t_{2}-t_{1}}$$

where *W*_1_ is the dry weight of the cotyledon-free seedling at radicle emergence (≈4 days post-inoculation, time *t*_1_) and *W*_2_ is the dry weight of the cotyledon-free seedling at the final harvest (12 days post-inoculation, time *t*_2_), giving Δ*t* ≈ 8 d.

### 4.11. Root Sampling and Image-Based Analysis of Root System Architecture

Roots were sampled at BBCH stage 12 (two fully expanded true leaves; 12 days after inoculation). Seedlings were carefully removed from the containers and roots were gently separated from the cotton substrate to minimize fine-root loss. Root systems were washed with low-pressure water over a 250 µm mesh to preserve fine-root structures, following published recommendations for root recovery and cleaning prior to image-based phenotyping [74,75]. Clean roots were spread in an A3 transparent tray filled with distilled water to minimize overlap and scanned on an Epson Expression 11000XL scanner (Seiko Epson Corporation, Suwa, Nagano, Japan) with cold-light LED illumination; scale calibration was verified with a 1 mm copper wire after every 15 samples.

Digitized images were analyzed with WinRHIZO (Regent Instruments Inc., Québec City, QC, Canada), quantifying total root length, projected root area, root surface area, average root diameter, and root volume, using 6 biological replicates per treatment.

### 4.12. Biochemical Determinations in Seedling Tissues

Seedlings were harvested 12 days after inoculation (BBCH stage 12), separated into roots and leaves, flash-frozen in liquid nitrogen, and stored at −80 °C until analysis; all tissue was ground under liquid nitrogen immediately before extraction. Determinations were performed separately for roots and leaves, using 4 biological replicates per treatment and organ, and results are expressed on a dry weight basis.

#### 4.12.1. Proline

Proline was determined as an indicator of osmotic adjustment following Bates et al. [76]. Approximately 100 mg of ground tissue was homogenized in 2 mL of 3% (*w*/*v*) sulfosalicylic acid and centrifuged; the supernatant was reacted with acid ninhydrin and glacial acetic acid (1 h, 100 °C), cooled on ice, and the chromophore extracted into toluene. Absorbance of the organic phase was read at 520 nm and quantified against an L-proline curve (µmol g^−1^ FW).

#### 4.12.2. Lipid Peroxidation

Lipid peroxidation was estimated as thiobarbituric acid reactive substances following Hodges et al. [77], whose correction accounts for interfering sugars. Absorbance was recorded at 532, 600, and 440 nm to correct for non-specific turbidity and sugar–TBARS complexes, and malondialdehyde (nmol MDA equiv. g^−1^ FW).

#### 4.12.3. Antioxidant Capacity (DPPH)

Antioxidant capacity was determined using the 2,2-diphenyl-1-picrylhydrazyl radical-scavenging assay (DPPH), following Chinnici et al. [78], with minor modifications. Dried plant tissue was ground and homogenized in 80% (*v*/*v*) methanol. The extracts were centrifuged at 13,000× *g* for 5 min at 4 °C, and 10 µL of the supernatant was used for the reaction with the DPPH reagent. Absorbance was measured at 515 nm using a UV–vis spectrophotometer (Thermo Scientific Spectronic Genesys 10 UV–vis Scanning, Madison, WI, USA). Antioxidant capacity was calculated against a Trolox calibration curve and expressed as Trolox equivalents g^−1^ FW, according to the tissue basis used for extraction.

#### 4.12.4. Total Phenolic and Flavonoid Contents

Total phenolic content was determined using the Folin–Ciocalteu method, following Slinkard and Singleton [79], with chlorogenic acid as the calibration standard. Briefly, ground leaf tissue was homogenized in 80% (*v*/*v*) methanol, and the extracts were centrifuged at 10,000× *g* for 5 min at 4 °C. Then, 10 µL of the supernatant was mixed with 100 µL of Folin–Ciocalteu reagent and incubated for 5 min. Absorbance was measured at 765 nm using a UV vis spectrophotometer (Thermo Scientific Spectronic Genesys 10 UV vis Scanning, Madison, WI, USA). Total phenolic content was calculated from a chlorogenic acid calibration curve and expressed as mg chlorogenic acid equivalents g^−1^ FW.

Total flavonoid content was determined using the aluminum chloride colorimetric method, following Zhishen et al. [80], with rutin as the calibration standard. Methanolic extracts were reacted with aluminum chloride reagent, and absorbance was recorded at 510 nm. Flavonoid concentration was calculated from a rutin calibration curve and expressed as mg rutin equivalents g^−1^ FW.

### 4.13. Experimental Design and Statistical Analysis

Experiments were arranged as factorial designs with independent biological replicates; the factors, levels, and replication used for each assay are detailed below. The seedling experiment comprised three crossed factors: cultivar (2 levels: Manteca, Señorita), PEG-8000 concentration (4 levels: 0, 5, 10 and 15%, *w*/*v*) and inoculation (4 levels: non-inoculated control, *Bacillus haynesii* CamB6, *Microbacterium* sp. Se63, or their consortium), with six replicates per combination (2 × 4 × 4; *N* = 192), analyzed by three-way ANOVA (or its aligned-rank-transform equivalent where residuals remained heteroscedastic after transformation) with Tukey-adjusted post hoc comparisons. The germination biochemical assay (total phenolic content and DPPH radical-scavenging activity; Section 4.8) added sampling time (3 levels: 24, 48, and 72 h) as a fourth crossed factor to the same cultivar × PEG × inoculation structure, with three replicates per combination (2 × 4 × 4 × 3; *N* = 288); given the interpretive difficulty of a four-way design, higher-order interaction terms were decomposed into simple effects using estimated marginal means, and only interactions that reached significance are reported as such in the text, with the corresponding lower-order (main-effect and two-way) comparisons presented otherwise. In vitro PEG-8000 tolerance assay followed a 3 (bacterial treatment) × 5 (concentration) design, phosphate mobilization a 2 (bacterial treatment) × 4 (phosphorus source) design, and IAA production a one-factor design (3 levels), each with three biological replicates per combination. Statistical analyses were performed in R using RStudio version 2023.03.0. Type III ANOVA and Levene’s tests were conducted using the car package, with sum-to-zero contrasts, whereas residual normality was assessed using base R stats. Variables remaining heteroscedastic after transformation were analyzed using the aligned rank transform implemented in ARTool. Significant effects were resolved using estimated marginal means and Tukey-adjusted comparisons in emmeans. *p*-values are reported for all comparisons discussed in the text; partial eta-squared (calculated using effectsize) is additionally reported only for headline contrasts, and complementary Bayesian model comparisons were performed using BayesFactor. Pearson correlation coefficients were used to assess the bivariate association between total phenolic content and DPPH radical-scavenging activity. Confocal observations were interpreted qualitatively, whereas culturable root-associated counts were analyzed separately by cultivar, with PEG concentration and inoculation as fixed factors. Given the number of factors and endpoints involved, full ANOVA tables for all assays are provided as Supplementary Material; the main text reports only significant effects and their associated *p*-values. A significance threshold of α = 0.05 was used throughout.

### 4.14. Use of Generative Artificial Intelligence

During the preparation of this manuscript, the authors used an AI-based language assistant for English-language editing, reference formatting, and reformatting of the manuscript to journal submission guidelines. No generative AI tool was used to design the experiments, to generate, analyze, or interpret the experimental data, or to produce the figures or the scientific conclusions reported here. The authors have reviewed and edited the output and take full responsibility for the content of this publication.

## Figures and Tables

**Table 1:** Qualitative in vitro plant-growth-promoting and enzymatic trait screen of *Microbacterium* sp. Se63 and *Bacillus haynesii* CamB6.

ID	Strain	P Solub.	N_2_ Fix.	Lipase	Catalase	Protease	Amylase	Mucoid	HCN	Sidero-Phores	Ammonia
*Microbacterium* sp.	Se63	+++	+	+	−	−	+	+	−	++	+
*Bacillus* sp.	CamB6	−	++	−	++	++	+	++	++	−	++

P solub., phosphate solubilization on Pikovskaya agar; N_2_ fix., growth on nitrogen-free medium; Lipase, Catalase, Protease and Amylase, corresponding enzymatic activities; Mucoid, mucoid colony phenotype; HCN, hydrogen cyanide production. Scoring: “+++”, very high activity; “++”, moderate activity; “+”, low activity; “−”, no activity. Representative plate assays are shown in Figure S1.

## Data Availability

All relevant data have been provided as Tables and Figures in the text and Supplementary Materials.

## Supplementary Materials

The following supporting information can be downloaded at: https://www.mdpi.com/article/10.3390/plants15193004/s1. Figure S1: Representative plate assays underlying the qualitative plant-growth-promoting trait screen; Figure S2: Mean soluble phosphorus released from four phosphorus sources (heat-map summary); Figure S3: Representative pure-culture reference plates and root-wash dilution plates; Figure S4: Colony morphology of *Bacillus haynesii* CamB6 and *Microbacterium* sp. Se63 in pure culture; Figure S5: Growth of *Bacillus haynesii* CamB6, *Microbacterium* sp. Se63 and the CamB6 + Se63 co-culture in the common semi-synthetic medium and in the two strain-specific maintenance media; Table S1: Total phenolic content and DPPH radical-scavenging activity in germinating seeds; Table S2: Colony morphology and culture characteristics of both strains.

## References

[1] Tay K., Zapata N., Urrea C.A., Elazab A., Garriga M., León L. (2025). Screening terminal drought tolerance in dry bean genotypes and commercial bean cultivars in Chile. Agronomy.

[2] Kaushal M., Wani S.P. (2016). Plant-growth-promoting rhizobacteria: Drought stress alleviators to ameliorate crop production in drylands. Ann. Microbiol..

[3] Poudel M., Mendes R., Costa L.A.S., Bueno C.G., Meng Y., Folimonova S.Y., Garrett K.A., Martins S.J. (2021). The role of plant-associated bacteria, fungi, and viruses in drought stress mitigation. Front. Microbiol..

[4] Chieb M., Gachomo E.W. (2023). The role of plant growth promoting rhizobacteria in plant drought stress responses. BMC Plant Biol..

[5] Zamani F., Majnoun Hosseini N., Oveisi M., Arvin K., Rabieyan E., Torkaman Z., Rodriguez D. (2024). Rhizobacteria and phytohormonal interactions increase drought tolerance in *Phaseolus vulgaris* through enhanced physiological and biochemical efficiency. Sci. Rep..

[6] Mohamed H.I., Eid A.W., Khalaf K.E., Joseph K.K., Mohamed M.A., Mohamed M.M., Ghanaim A.M. (2026). Harnessing plant growth-promoting microorganisms to improve drought resilience in common bean (*Phaseolus vulgaris* L.). BMC Plant Biol..

[7] Del-Canto A., Sanz-Saez Á., Sillero-Martínez A., Mintegi E., Lacuesta M. (2023). Selected indigenous drought tolerant rhizobium strains as promising biostimulants for common bean in Northern Spain. Front. Plant Sci..

[8] Corretto E., Antonielli L., Sessitsch A., Höfer C., Puschenreiter M., Widhalm S., Swarnalakshmi K., Brader G. (2020). Comparative genomics of *Microbacterium* species to reveal diversity, potential for secondary metabolites and heavy metal resistance. Front. Microbiol..

[9] Gaete A., Mandakovic D., González M. (2020). Isolation and identification of soil bacteria from extreme environments of Chile and their plant beneficial characteristics. Microorganisms.

[10] Muñoz-Torres P., Márquez S.L., Sepúlveda-Chavera G., Cárdenas-Ninasivincha S., Arismendi-Macuer M., Huanca-Mamani W., Aguilar Y., Quezada A., Bugueño F. (2023). Isolation and identification of bacteria from three geothermal sites of the Atacama Desert and their plant-beneficial characteristics. Microorganisms.

[11] Karimi E., Aliasgharzad N., Esfandiari E., Hassanpouraghdam M.B., Neu T.R., Buscot F., Reitz T., Breitkreuz C., Tarkka M.T. (2022). Biofilm forming rhizobacteria affect the physiological and biochemical responses of wheat to drought. AMB Express.

[12] Santander C., González F., Pérez U., Ruiz A., Aroca R., Santos C., Cornejo P., Vidal G. (2024). Enhancing water status and nutrient uptake in drought-stressed lettuce plants (*Lactuca sativa* L.) via inoculation with different *Bacillus* spp. isolated from the Atacama Desert. Plants.

[13] Banerjee A., Mohammed Breig S.J., Gómez A., Sánchez-Arévalo I., González-Faune P., Sarkar S., Bandopadhyay R., Vuree S., Cornejo J., Tapia J. (2022). Optimization and characterization of a novel exopolysaccharide from *Bacillus haynesii* CamB6 for food applications. Biomolecules.

[14] Marín-Sanhueza C., Echeverría-Vega A., Gómez A., Cabrera-Barjas G., Romero R., Banerjee A. (2022). Stress dependent biofilm formation and bioactive melanin pigment production by a thermophilic *Bacillus* species from Chilean hot spring. Polymers.

[15] Núñez-Montero K., Quezada-Solís D., Khalil Z.G., Capon R.J., Andreote F.D., Barrientos L. (2020). Genomic and metabolomic analysis of Antarctic bacteria revealed culture and elicitation conditions for the production of antimicrobial compounds. Biomolecules.

[16] Saleem M., Nawaz F., Hussain M.B., Ikram R.M. (2021). Comparative effects of individual and consortia plant growth promoting bacteria on physiological and enzymatic mechanisms to confer drought tolerance in maize (*Zea mays* L.). J. Soil Sci. Plant Nutr..

[17] Yang X., Li Q., Zhang Y., He S., Li C., Xu X., Liu Y., Yao T. (2026). Construction of drought-resistant microbial consortium and effect on alfalfa growth under drought stress. Plants.

[18] Massucato L.R., Silva M.B., Mosela M., Watanabe L.S., Afonso L., Marcos A.W., Nogueira A.F., de Sousa N.V., Fendrich R.C., Faria M.V. (2025). Enzymatic activity and organic acid profile of phosphate-solubilizing bacterial inoculants and their agronomic effectiveness in soybean. Microorganisms.

[19] Long H., Wasaki J. (2023). Effects of phosphate-solubilizing bacteria on soil phosphorus fractions and supply to maize seedlings grown in lateritic red earths and cinnamon soils. Microbes Environ..

[20] Faller L., Leite M.F.A., Kuramae E.E. (2024). Enhancing phosphate-solubilising microbial communities through artificial selection. Nat. Commun..

[21] Collavino M.M., Sansberro P.A., Mroginski L.A., Aguilar O.M. (2010). Comparison of in vitro solubilization activity of diverse phosphate-solubilizing bacteria native to acid soil and their ability to promote *Phaseolus vulgaris* growth. Biol. Fertil. Soils.

[22] Raymond N.S., Gómez-Muñoz B., van der Bom F.J.T., Nybroe O., Jensen L.S., Müller-Stöver D.S., Oberson A., Richardson A.E. (2021). Phosphate-solubilising microorganisms for improved crop productivity: A critical assessment. New Phytol..

[23] Elhaissoufi W., Khourchi S., Ibnyasser A., Ghoulam C., Rchiad Z., Zeroual Y., Lyamlouli K., Bargaz A. (2020). Phosphate solubilizing rhizobacteria could have a stronger influence on wheat root traits and aboveground physiology than rhizosphere P solubilization. Front. Plant Sci..

[24] Ertürk Y., Erçişli S., Haznedar A., Çakmakçı R. (2010). Effects of plant growth promoting rhizobacteria (PGPR) on rooting and root growth of kiwifruit (*Actinidia deliciosa*) stem cuttings. Biol. Res..

[25] Ganesh J., Hewitt K., Devkota A.R., Wilson T., Kaundal A. (2024). IAA-producing plant growth promoting rhizobacteria from Ceanothus velutinus enhance cutting propagation efficiency and Arabidopsis biomass. Front. Plant Sci..

[26] Buqori D.M.A.I., Sugiharto B., Suherman, Siswoyo T.A., Hariyono K. (2025). Mitigating drought stress by application of drought-tolerant *Bacillus* spp. enhanced root architecture, growth, antioxidant and photosynthetic genes expression in sugarcane. Sci. Rep..

[27] Mahreen N., Yasmin S., Asif M., Yahya M., Ejaz K., Mehboob-ur-Rahman, Yousaf S., Amin I., Zulfiqar S., Imran A. (2023). Mitigation of water scarcity with sustained growth of rice by plant growth promoting bacteria. Front. Plant Sci..

[28] Prasanna Kumar B., Trimurtulu N., Vijaya Gopal A., Nagaraju Y. (2022). Impact of culturable endophytic bacteria on soil aggregate formation and peanut (Arachis hypogaea L.) growth and yield under drought conditions. Curr. Microbiol..

[29] Burman E., Bengtsson-Palme J. (2021). Microbial community interactions are sensitive to small changes in temperature. Front. Microbiol..

[30] Díaz P.R., Merlo F., Carrozzi L., Valverde C., Creus C.M., Maroniche G.A. (2023). Lettuce growth improvement by *Azospirillum argentinense* and fluorescent *Pseudomonas* co-inoculation depends on strain compatibility. Appl. Soil Ecol..

[31] Santiago C.D., Yagi S., Ijima M., Nashimoto T., Sawada M., Ikeda S., Asano K., Orikasa Y., Ohwada T. (2017). Bacterial compatibility in combined inoculations enhances the growth of potato seedlings. Microbes Environ..

[32] Shao J., Liu Y., Xie J., Štefanič P., Lv Y., Fan B., Mandic-Mulec I., Zhang R., Shen Q., Xu Z. (2022). Annulment of bacterial antagonism improves plant beneficial activity of a *Bacillus velezensis* consortium. Appl. Environ. Microbiol..

[33] Pineda-Pineda J.J., Muñoz-Rojas J., Morales-García Y.E., Trenado-Sánchez E., Sigarreta J.M. (2025). Quantification and determination of compatible bacterial consortia. Microb. Biotechnol..

[34] Prigigallo M.I., Staropoli A., Vinale F., Bubici G. (2023). Interactions between plant-beneficial microorganisms in a consortium: *Streptomyces microflavus* and *Trichoderma harzianum*. Microb. Biotechnol..

[35] Thomas-Barry G., Martin C.S., Ramsubhag A., Eudoxie G., Miller J.R. (2024). Multi-trait efficiency and interactivity of bacterial consortia used to enhance plant performance under water stress conditions. Microbiol. Res..

[36] Fiodor A., Ajijah N., Dziewit Ł., Pranaw K. (2023). Biopriming of seed with plant growth-promoting bacteria for improved germination and seedling growth. Front. Microbiol..

[37] Jakubowska Z., Gradowski M., Dobrzyński J. (2025). Role of plant growth-promoting bacteria (PGPB) in enhancing phenolic compounds biosynthesis and its relevance to abiotic stress tolerance in plants: A review. Antonie Van Leeuwenhoek.

[38] Jeon J.-S., Rybka D., Carreno-Quintero N., De Vos R., Raaijmakers J.M., Etalo D.W. (2022). Metabolic signatures of rhizobacteria-induced plant growth promotion. Plant Cell Environ..

[39] Caretto S., Linsalata V., Colella G., Mita G., Lattanzio V. (2015). Carbon fluxes between primary metabolism and phenolic pathway in plant tissues under stress. Int. J. Mol. Sci..

[40] Monson R.K., Trowbridge A.M., Lindroth R.L., Lerdau M.T. (2022). Coordinated resource allocation to plant growth–defense tradeoffs. New Phytol..

[41] Tong Z., Tao Z., Li F., He J., Qin S. (2026). Multi-omics integration reveals temporal partitioning between metabolic priming and proliferative expansion in PGPR-treated cherry plants. Int. J. Mol. Sci..

[42] Gao C., Xu L., Montoya L., Madera M., Hollingsworth J., Chen L., Purdom E., Singan V., Vogel J., Hutmacher R.B. (2022). Co-occurrence networks reveal more complexity than community composition in resistance and resilience of microbial communities. Nat. Commun..

[43] Asghar M.J., Tariq M., Hameed S., Akhtar K.P., Ghaffar M., Shahid M., Shah T.M. (2025). Assessing the impact of PGPR consortia inoculum on mung bean [*Vigna radiata* (L.) Wilczek] mutant varieties in different soil types. Plant Breed..

[44] Remans R., Beebe S., Blair M., Manrique G., Tovar E., Rao I., Croonenborghs A., Torres-Gutierrez R., El-Howeity M., Michiels J. (2008). Physiological and genetic analysis of root responsiveness to auxin-producing plant growth-promoting bacteria in common bean (*Phaseolus vulgaris* L.). Plant Soil.

[45] Kuzmicheva Y.V., Shaposhnikov A.I., Petrova S.N., Makarova N.M., Tychinskaya I.L., Puhalsky J.V., Parahin N.V., Tikhonovich I.A., Belimov A.A. (2017). Variety specific relationships between effects of rhizobacteria on root exudation, growth and nutrient uptake of soybean. Plant Soil.

[46] Pacheco-Moreno A., Bollmann-Giolai A., Chandra G., Brett P., Davies J., Thornton O., Poole P., Ramachandran V., Brown J.K.M., Nicholson P. (2024). The genotype of barley cultivars influences multiple aspects of their associated microbiota via differential root exudate secretion. PLoS Biol..

[47] Duan B., Li L., Chen G., Su-Zhou C., Li Y., Merkeryan H., Liu W., Liu X. (2021). 1-Aminocyclopropane-1-carboxylate deaminase-producing plant growth-promoting rhizobacteria improve drought stress tolerance in grapevine (*Vitis vinifera* L.). Front. Plant Sci..

[48] Canavera G., Bellotti G., Tiwari H., Frioni T., Puglisi E. (2026). Drought-tolerant rhizobacterial consortia enhance grapevine growth and tolerance to water deficit. Front. Plant Sci..

[49] Xu L., Dong Z., Chiniquy D., Pierroz G., Deng S., Gao C., Diamond S., Simmons T., Wipf H.M.-L., Caddell D. (2021). Genome-resolved metagenomics reveals role of iron metabolism in drought-induced rhizosphere microbiome dynamics. Nat. Commun..

[50] Kylyshbayeva G., Bishimbayeva N., Jatayev S., Eliby S., Shavrukov Y. (2025). Polyethylene glycol (PEG) application triggers plant dehydration but does not accurately simulate drought. Plants.

[51] Töpfer V., Melzer M., Snowdon R.J., Stahl A., Matros A., Wehner G. (2024). PEG treatment is unsuitable to study root related traits as it alters root anatomy in barley (*Hordeum vulgare* L.). BMC Plant Biol..

[52] Gupta S., Pandey S. (2019). ACC deaminase producing bacteria with multifarious plant growth promoting traits alleviates salinity stress in French bean (*Phaseolus vulgaris*) plants. Front. Microbiol..

[53] Nautiyal C.S. (1999). An efficient microbiological growth medium for screening phosphate solubilizing microorganisms. FEMS Microbiol. Lett..

[54] Mehta S., Nautiyal C.S. (2001). An efficient method for qualitative screening of phosphate-solubilizing bacteria. Curr. Microbiol..

[55] Bautista-Cruz A., Antonio-Revuelta B., Martínez Gallegos V.d.C., Báez-Pérez A. (2019). Phosphate-solubilizing bacteria improve Agave angustifolia Haw. growth under field conditions. J. Sci. Food Agric..

[56] Gordon S.A., Weber R.P. (1951). Colorimetric estimation of indoleacetic acid. Plant Physiol..

[57] Michel B.E. (1983). Evaluation of the water potentials of solutions of polyethylene glycol 8000 both in the absence and presence of other solutes. Plant Physiol..

[58] Michel B.E., Kaufmann M.R. (1973). The osmotic potential of polyethylene glycol 6000. Plant Physiol..

[59] Meza C., Valenzuela F., Echeverría-Vega A., Gomez A., Sarkar S., Cabeza R.A., Arencibia A.D., Quiroz K., Carrasco B., Banerjee A. (2022). Plant-growth-promoting bacteria from rhizosphere of Chilean common bean ecotype (*Phaseolus vulgaris* L.) supporting seed germination and growth against salinity stress. Front. Plant Sci..

[60] Pagano A., Araújo S.d.S., Macovei A., Leonetti P., Balestrazzi A. (2017). The seed repair response during germination: Disclosing correlations between DNA repair, antioxidant response, and chromatin remodeling in Medicago truncatula. Front. Plant Sci..

[61] Singleton V.L., Rossi J.A. (1965). Colorimetry of total phenolics with phosphomolybdic-phosphotungstic acid reagents. Am. J. Enol. Vitic..

[62] Flores-Castañón N., Meza C., Valenzuela F., Ormeño C., Tapia J., Carrasco B., Banerjee A. (2025). Effect of plant growth-promoting bacteria (PGPB) on the antioxidant and antibacterial activities in Chilean ‘sapito’ common beans. BMC Plant Biol..

[63] Mhada M., Zvinavashe A.T., Hazzoumi Z., Zeroual Y., Marelli B., Kouisni L. (2021). Bioformulation of silk-based coating to preserve and deliver Rhizobium tropici to *Phaseolus vulgaris* under saline environments. Front. Plant Sci..

[64] Bernate I., Sabovics M., Tomsone L. (2020). Influence of germination conditions and parameters on the content of total phenolic compounds and scavenging activity in germinated seeds. Rural Sustain. Res..

[65] Eum H.L., Park Y., Yi T.G., Lee J.W., Ha K.-S., Choi I.-Y., Park N.I. (2020). Effect of germination environment on the biochemical compounds and anti-inflammatory properties of soybean cultivars. PLoS ONE.

[66] Brand-Williams W., Cuvelier M.E., Berset C. (1995). Use of a free radical method to evaluate antioxidant activity. LWT–Food Sci. Technol..

[67] Anand A., Kumari A., Thakur M., Koul A. (2019). Hydrogen peroxide signaling integrates with phytohormones during the germination of magnetoprimed tomato seeds. Sci. Rep..

[68] Rangjaroen C., Lumyong S., Sloan W.T., Sungthong R. (2019). Herbicide-tolerant endophytic bacteria of rice plants as the biopriming agents for fertility recovery and disease suppression of unhealthy rice seeds. BMC Plant Biol..

[69] Barillot C.D.C., Sarde C.-O., Bert V., Tarnaud E., Cochet N. (2013). A standardized method for the sampling of rhizosphere and rhizoplan soil bacteria associated to a herbaceous root system. Ann. Microbiol..

[70] Majeed A., Abbasi M.K., Hameed S., Imran A., Rahim N. (2015). Isolation and characterization of plant growth-promoting rhizobacteria from wheat rhizosphere and their effect on plant growth promotion. Front. Microbiol..

[71] Carroll D., Holden N., Gifford M.L., Dupuy L.X. (2020). Framework for quantification of the dynamics of root colonization by Pseudomonas fluorescens isolate SBW25. Front. Microbiol..

[72] Korir H., Mungai N.W., Thuita M., Hamba Y., Masso C. (2017). Co-inoculation effect of rhizobia and plant growth promoting rhizobacteria on common bean growth in a low phosphorus soil. Front. Plant Sci..

[73] Sarkar S., Echeverría-Vega A., Banerjee A., Bandopadhyay R. (2021). Decolourisation and biodegradation of textile di-azo dye Congo red by Chryseobacterium geocarposphaerae DD3. Sustainability.

[74] Wang M.-B., Zhang Q. (2009). Issues in using the WinRHIZO system to determine physical characteristics of plant fine roots. Acta Ecol. Sin..

[75] Matzner S.L., Rettedal D.D., Harmon D.A., Beukelman M.R. (2014). Constraints to hydraulic acclimation under reduced light in two contrasting *Phaseolus vulgaris* cultivars. J. Exp. Bot..

[76] Bates L.S., Waldren R.P., Teare I.D. (1973). Rapid determination of free proline for water-stress studies. Plant Soil.

[77] Hodges D.M., DeLong J.M., Forney C.F., Prange R.K. (1999). Improving the thiobarbituric acid-reactive-substances assay for estimating lipid peroxidation in plant tissues containing anthocyanin and other interfering compounds. Planta.

[78] Chinnici F., Bendini A., Gaiani A., Riponi C. (2004). Radical scavenging activities of peels and pulps from cv. Golden Delicious apples as related to their phenolic composition. J. Agric. Food Chem..

[79] Slinkard K., Singleton V.L. (1977). Total phenol analysis: Automation and comparison with manual methods. Am. J. Enol. Vitic..

[80] Jia Z., Tang M., Wu J. (1999). The determination of flavonoid contents in mulberry and their scavenging effects on superoxide radicals. Food Chem..

