# Supplementary material for: Extremophilic Bacteria Improve Germination, Early Vigor, and Oxidative-Stress Status in Phaseolus vulgaris Under Simulated Water Deficit
Source: Plants (Basel). 2026 Sep 30;15(19):3004. doi: 10.3390/plants15193004 (PMC13666678; doi:10.3390/plants15193004)
Supplement: Supplementary file 1 [file plants-15-03004-s001.zip › plants-4536884-supplementary.pdf]

Supplementary Materials

Extremophilic Bacteria Improve Germination, Early Vigor, and Oxidative Stress Status in *Phaseolus vulgaris* under Simulated Water Deficit

Rodrigo Mora-Sanhueza, Cynthia Meza, Nicolas Flores, Ricardo Tighe-Neira, Aparna Bernarjee, Kattia Núñez-Montero, Valentina Vallejos, Patricio Arce-Johnson and Claudio Inostroza-Blancheteau

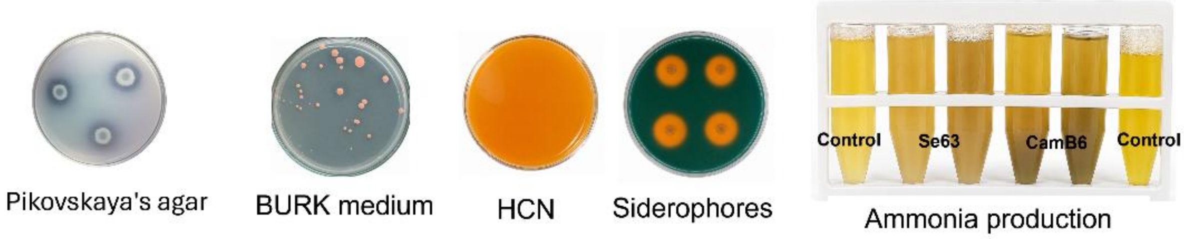

Figure S1. Representative plate assays underlying the qualitative plant-growth-promoting trait screen of *Microbacterium* sp. Se63 and *Bacillus haynesii* CamB6 (Table 1). From left to right: phosphate solubilization halos on Pikovskaya agar; colony growth on nitrogen-free BURK medium; hydrogen cyanide production on King's B agar with glycine; siderophore production on Chrome Azurol S agar; and ammonia production in peptone water after addition of Nessler's reagent (non-inoculated controls at both ends, Se63 and CamB6 in between). Images are representative of three biological replicates. [This panel was previously embedded inside the Table 1 image; the strain identity of each individual plate should be confirmed by the authors and stated explicitly in the legend.]

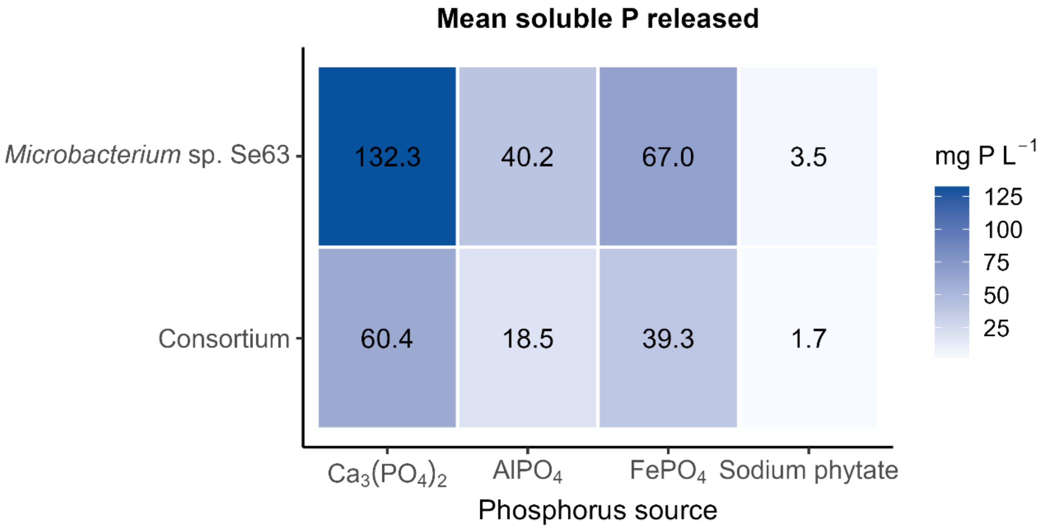

Figure S2. Mean soluble phosphorus released by *Microbacterium* sp. Se63 and the CamB6 + Se63 consortium from four phosphorus sources (tricalcium phosphate, aluminium phosphate, iron phosphate and sodium phytate) in NBRIP liquid medium. Heat-map summary of treatment means (mg P L^{-1}; n = 3) corresponding to the distributions shown in Figure 1. *Bacillus haynesii* CamB6 alone was not included in this quantitative assay, as the qualitative screen (Table 1) had already shown it lacks detectable phosphate-solubilizing activity.

Table S1. Total phenolic content (TPC) and DPPH radical-scavenging activity in germinating seeds of *Phaseolus vulgaris* cvs. Manteca and Señorita under bacterial inoculation (non-inoculated control, CamB6, Se63, two-strain consortium "Both") and PEG-8000-induced osmotic stress (0, 5, 10 and 15%), at 24, 48, 72 and 92 h post-imbibition. Data are treatment means (n = 3); within each Cultivar × Time × PEG-8000 combination and variable, means followed by different lowercase

letters differ significantly (four-way factorial ANOVA, Tukey's HSD, $\alpha = 0.05$). [Note: this table reports four sampling times (24, 48, 72 and 92 h), whereas Section 4.13 of the main text describes three (24, 48 and 72 h) and N = 288; the design description and this table must be reconciled before submission.]

Cultivar	Time (h)	PEG-8000 (%)	Total phenolic content (mg GAE g^{-1} FW)				DPPH radical-scavenging activity (% RSA)			
			Control	CamB6	Se63	Both	Control	CamB6	Se63	Both
Manteca	24	0	8.23 c	11.23 ab	9.50 bc	11.67 a	30.00 b	38.00 a	36.00 a	40.00 a
Manteca	24	5	8.47 c	10.77 b	10.90 b	12.60 a	31.00 b	39.00 a	38.00 a	42.00 a
Manteca	24	10	8.43 c	11.07 b	10.67 b	13.43 a	28.00 b	36.00 a	35.00 a	39.00 a
Manteca	24	15	8.73 d	11.73 b	10.10 c	14.53 a	23.00 b	30.00 a	29.00 a	33.00 a
Manteca	48	0	27.40 a	24.77 ab	23.80 b	21.73 b	42.00 b	52.00 a	50.00 a	55.00 a
Manteca	48	5	28.03 a	27.03 ab	24.10 b	24.57 ab	43.00 b	53.00 a	52.00 a	56.00 a
Manteca	48	10	31.17 a	25.97 b	27.00 b	26.57 b	38.00 b	49.00 a	47.00 a	52.00 a
Manteca	48	15	32.33 a	29.70 b	29.57 b	27.27 c	30.00 b	40.00 a	39.00 a	42.00 a
Manteca	72	0	14.00 a	11.27 ab	11.33 ab	9.23 b	40.00 a	37.00 ab	36.00 b	30.33 c
Manteca	72	5	14.53 a	13.23 ab	11.50 c	12.10 bc	39.00 a	34.33 ab	29.33 b	35.33 a
Manteca	72	10	15.60 a	13.77 a	13.93 a	13.77 a	41.00 a	33.33 b	34.00 b	30.33 b
Manteca	72	15	16.83 a	15.20 b	14.43 b	14.00 b	44.67 a	37.00 b	32.67 b	33.00 b
Manteca	92	0	10.17 a	8.50 ab	7.77 b	6.43 b	38.33 a	29.67 b	33.00 b	30.00 b
Manteca	92	5	10.57 a	10.17 a	8.07 a	8.33 a	40.00 a	36.00 b	32.00 c	31.00 c
Manteca	92	10	12.90 a	9.50 b	10.10 b	9.83 b	41.00 a	33.33 bc	36.33 b	31.00 c
Manteca	92	15	13.73 a	11.77 bc	11.90 b	10.27 c	44.67 a	39.00 b	38.67 b	33.33 c
Señorita	24	0	10.00 a	8.60 b	8.80 ab	8.00 b	23.33 c	32.67 b	28.00 bc	39.67 a
Señorita	24	5	11.00 a	8.62 b	9.30 b	8.20 b	24.00 c	31.33 b	32.67 b	41.33 a
Señorita	24	10	12.00 a	10.00 b	9.80 bc	8.50 c	24.33 c	33.67 ab	32.00 b	40.33 a
Señorita	24	15	13.00 a	10.50 b	10.50 b	9.00 c	23.33 c	33.00 b	30.00 b	42.00 a
Señorita	48	0	16.00 a	13.97 b	14.00 b	12.50 c	51.00 b	55.67 ab	59.00 a	51.00 b
Señorita	48	5	17.00 a	15.00 b	14.50 b	13.00 c	54.33 a	59.00 a	58.00 a	57.33 a
Señorita	48	10	18.00 a	15.50 b	15.00 b	13.50 c	55.33 a	51.33 a	53.33 a	51.33 a
Señorita	48	15	19.00 a	16.00 b	15.50 b	14.00 c	57.33 ab	59.67 a	53.67 b	53.33 b

Cultivar	Time (h)	PEG-8000 (%)	Total phenolic content (mg GAE g⁻¹ FW)				DPPH radical-scavenging activity (% RSA)			
			Control	CamB6	Se63	Both	Control	CamB6	Se63	Both
Señorita	72	0	13.50 a	9.33 b	11.23 ab	10.00 b	49.67 a	41.33 ab	39.67 ab	34.33 b
Señorita	72	5	14.00 a	11.07 a	12.10 a	11.87 a	52.67 a	47.00 ab	40.33 b	42.00 b
Señorita	72	10	14.80 a	13.20 ab	12.73 b	11.70 b	56.00 a	48.33 ab	47.67 b	47.00 b
Señorita	72	15	15.23 a	14.20 b	13.93 b	13.50 b	59.67 a	55.67 ab	50.00 bc	47.00 c
Señorita	92	0	12.00 a	10.57 b	10.90 b	10.37 b	37.67 a	30.33 ab	29.00 b	23.67 b
Señorita	92	5	12.27 a	10.00 ab	11.07 ab	9.67 b	39.33 a	35.67 ab	27.33 c	28.67 bc
Señorita	92	10	13.43 a	11.17 b	11.77 b	10.93 b	47.00 a	32.33 b	33.67 b	30.33 b
Señorita	92	15	14.47 a	12.77 b	12.70 b	11.23 c	49.00 a	39.00 b	39.00 b	33.00 b

GAE, gallic acid equivalents; DPPH, 2,2-diphenyl-1-picrylhydrazyl; RSA, radical-scavenging activity relative to a methanol blank.

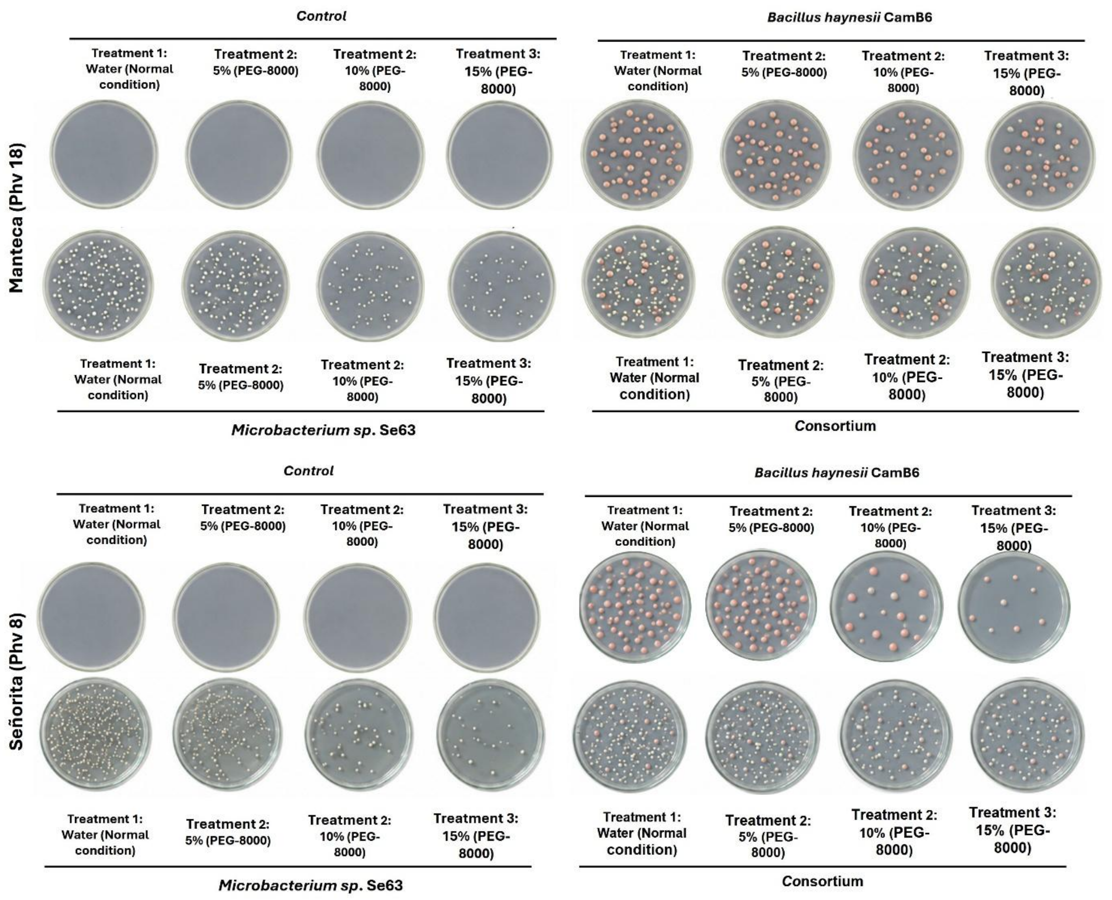

Figure S3. Representative pure-culture reference plates and root-wash dilution plates for cv. Manteca (Phv 18) and cv. Señorita (Phv 8), by inoculation treatment (non-inoculated control, *Bacillus haynesii* CamB6, *Microbacterium* sp. Se63, and the two-strain consortium) and PEG-8000 level (0, 5, 10 and 15%), 7 days post-inoculation. Underlying photographic record for the colony counts summarized in Figure 5.

Table S2. Colony morphology and culture characteristics of *Microbacterium* sp. Se63 and *Bacillus haynesii* CamB6. [Caption drafted during reformatting; authors should confirm the wording and, if the item is retained, verify that "Sintetic" is corrected to "semi-synthetic medium" and that the growth temperature range given for CamB6 (20–60 °C) is intended.]

ID	Strain	Border	Elevation	Form	Opaque	Translucent	Opalescent	Brilliant	Pigmentation	Common medium	Isolated medium	Growth temp (°C)	Growth time (days)	Growth rate	Isolation site & by whom
Microbacterium sp.	Se63	Integer	Plana	Round	–	semi-translucent	–	Yes	salmon-pink (pastel pink/coral).	Sintetic	ISP2	10–35	2–3	Normal	Soil
Bacillus sp.	CamB6	Integer	Convex	Round	Yes	–	–	Yes	cream to translucent white (off-white)	Sintetic	Jensen	20-60	2–4	Normal	Thermal water

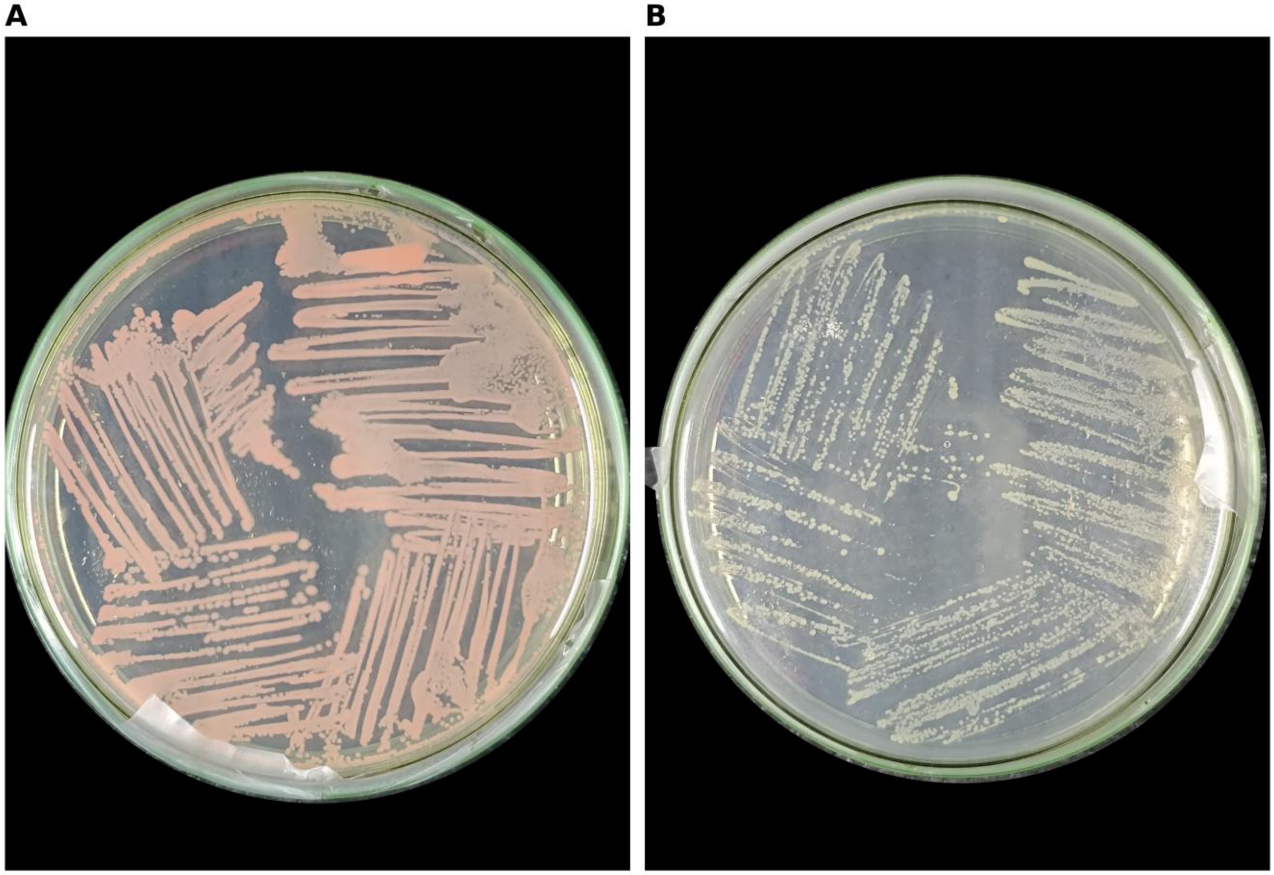

Figure S4. Colony morphology of *Bacillus haynesii* CamB6 and *Microbacterium* sp. Se63 in pure culture on solid semi-synthetic medium after 48 h at 27 °C. CamB6 forms pigmented (orange-salmon), convex, round colonies with an entire margin; Se63 forms cream to translucent white, flat, round colonies with an entire margin. These morphotypes are the basis for the presumptive differentiation of the two partners on root-wash plates (Figure 5A). Descriptive characteristics are tabulated in Table S2. [Authors: confirm incubation time and that the described pigmentation matches the images inserted above.]

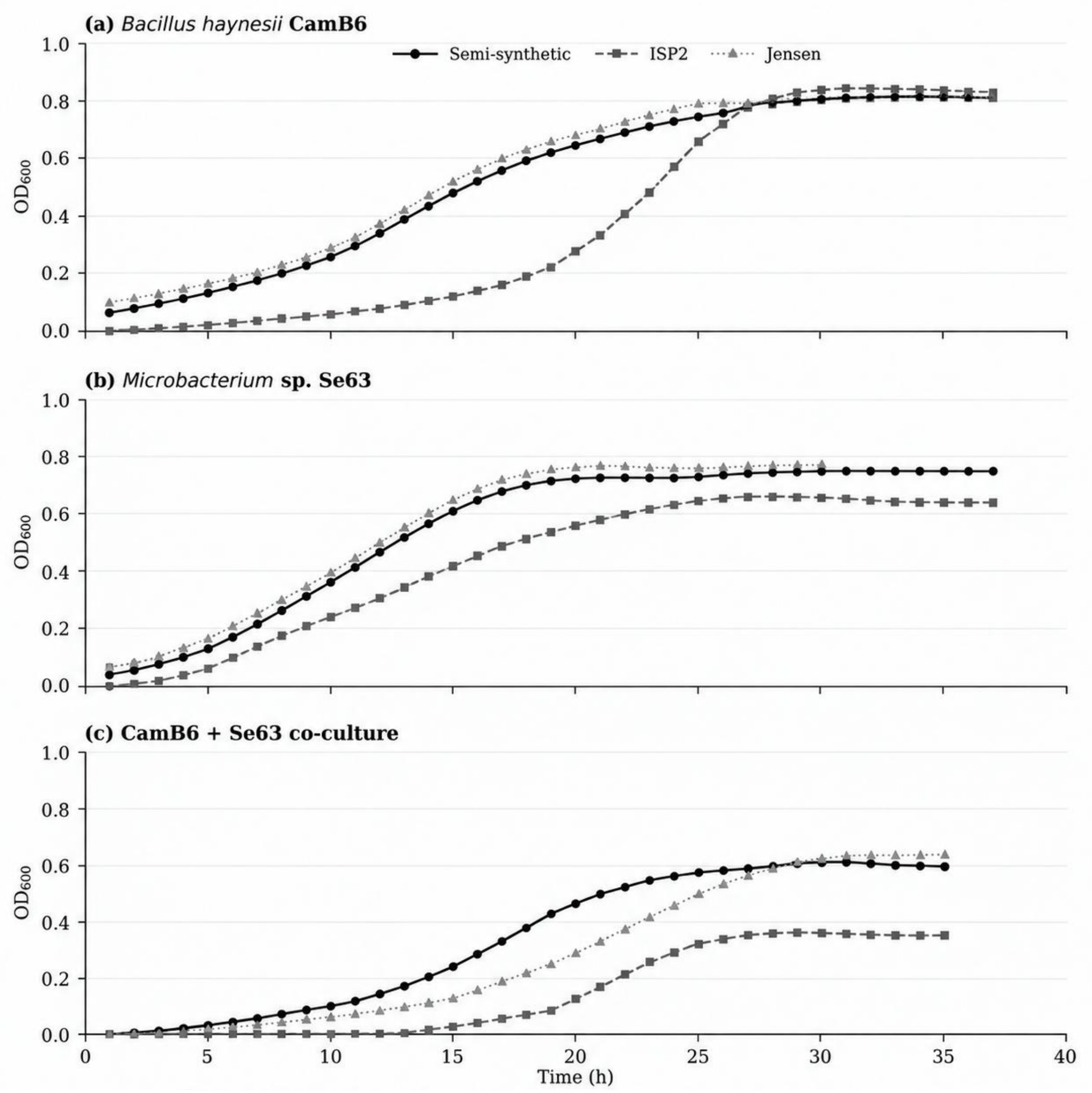

Figure S5. Growth of (a) *Bacillus haynesii* CamB6, (b) *Microbacterium* sp. Se63 and (c) the CamB6 + Se63 co-culture in the common semi-synthetic medium used for all comparative assays and in the two strain-specific maintenance media (Jensen and ISP2). Cultures were incubated at 27 °C and 150 rpm and optical density at 600 nm was recorded hourly (n = 3). All three cultures grow in the shared semi-synthetic medium at the common incubation temperature, which is the operational requirement for co-cultivation. Se63 enters exponential growth from about 5 h and plateaus near OD600 0.62 by 28 h, whereas CamB6 shows a longer lag, rises steeply between 15 and 28 h and plateaus near OD600 0.80. The co-culture follows a regular sigmoidal trajectory and reaches a stable stationary phase near OD600 0.60 by 29 h, and does so best in the semi-synthetic medium, growing more slowly in Jensen medium and reaching only about OD600 0.36 in ISP2. Media are distinguished by marker shape and line style so that the figure remains legible in greyscale.
